# Cross-cultural Adaptation of the Adverse Childhood Experiences International Questionnaire

**DOI:** 10.11606/s1518-8787.2021055003140

**Published:** 2021-11-18

**Authors:** Flávia Garcia Pereira, Maria Carmen Viana

**Affiliations:** I Universidade Federal do Espírito Santo Vitória ES Brasil Universidade Federal do Espírito Santo. Programa de Pós-Graduação em Saúde Coletiva. Vitória, ES, Brasil; II Universidade Federal do Espírito Santo Departamento de Medicina Social Vitória ES Brasil Universidade Federal do Espírito Santo. Departamento de Medicina Social. Vitória, ES, Brasil

**Keywords:** Adverse Childhood Experiences, Surveys and Questionnaires, Translations, Adult Survivors of Child Adverse Events, Cross-Cultural Comparison

## Abstract

**OBJECTIVE:**

To translate, adapt and validate the contents of the Adverse Childhood Experiences International Questionnaire to a Portuguese version, to be used in Brazil, named *Questionário Internacional de Experiências Adversas na Infância.*

**METHODS:**

This is a methodological study of cross-cultural adaptation of evaluation instruments presenting the results of semantic equivalence between the original instrument and the adapted version. The semantic equivalence of the instrument involved the following steps: 1) two translations and a synthesis of the translations; 2) two retranslations; 3) validation of contents by eight health workers; 4) synthesis of the retranslations; 5) pre-tests to assess acceptability, understanding and emotional impact of the questions; and, finally, 6) writing of the final version of the instrument.

**RESULTS:**

the adapted version proved to be easy to apply and to understand and achieved good semantic equivalence when compared to the original version. The psychometric properties of the instrument still need to be evaluated. Limitations and recommendations for improving the instrument and its use are presented.

**CONCLUSION:**

The process of cross-cultural adaptation of the Adverse Childhood Experiences International Questionnaire resulted in an adapted version to Brazilian Portuguese.

## INTRODUCTION

Although the history and documentation of the occurrence of child abuse is not recent^1,2^, the systematic study of adverse childhood experiences (ACE) has grown in interest in the scientific community since the 1990s, after the publication of the results of the Adverse Childhood Experiences (ACE) Study^3,4^.

The *ACE Study*, conducted in San Diego by the Department of Preventive Medicine for Kaiser Permanente^®^ in collaboration with the United States Centers for Disease Control and Prevention (CDC), is considered a milestone in investigation of exposure to child abuse and neglect as a risk factor associated with negative health and wellness outcomes throughout life^3,4^. Since its publication, several epidemiological studies have been conducted, emphasizing a growing worldwide concern about the consequences of child abuse, not only immediately, but also in the long term^5–7^.

Adverse childhood experiences can result in morbidity and mortality directly due to violence, though the consequences more often involve emotional, cognitive and behavioral damage that increases the risk of both the development of chronic diseases in adulthood and exposure to risk behaviors in adolescence^8^. In this sense, several studies have shown positive and dose-dependent associations between exposure to ACE and coronary heart disease, diabetes, cancer, chronic obstructive pulmonary disease^3^, mental disorders^9–11^, chronic fatigue syndrome^12^, chronic pain^13^, suicide attempts^14^, risky sexual behavior^15^, early use of alcohol, tobacco and other drugs^16,17^, and premature mortality^18,19^, among others. Furthermore, negative outcomes in school life, income and occupation have been described^20^.

A better understanding of the harmful role of ACE can support the direction and implementation of adequate prevention, intervention and treatment strategies, both in the creation of public health, justice and civic policies and in raising awareness, better training and integration of workers involved in fighting ill-treatment in children and adolescents and its consequences^21^. In this sense, the creation of standardized ACE evaluation instruments is crucial for reliable and valid measures to improve the quality of information.

In the last three decades, several instruments for investigation of ACE have been proposed, encompassing the evaluation of different categories and environmental contexts, with insufficient subsequent verification of their psychometric properties^22–24^. We should note that until this article was written, no instruments developed in Brazil had been found, although some have been adapted for use in Brazilian populations^25,26^ The wide variety of instruments used has made it difficult to compare the results produced, not only between diverse social contexts, but also between different periods in the same population. In this perspective, the World Health Organization (WHO), in collaboration with the CDC, developed the Adverse Childhood Experiences International Questionnaire (ACE-IQ) to provide a standardized instrument for worldwide evaluation and surveillance of ACE and to enable the creation of worldwide child abuse prevention programs and policies^27,28^.

The ACE-IQ was produced to be applied to adults (≥ 18 years) with the goal of identifying and evaluating prior exposure to 13 different categories of ACE, including sexual, emotional and physical abuse, emotional and physical neglect, family violence, alcohol and drug use, mental illness or suicide in the home, family involvement with criminal activity, parental separation or divorce, community violence, collective violence and bullying^27,28^. It is important to highlight that all ACE research instruments collect retrospective information, subject to memory biases; in addition, survival bias should be considered since serious violence of different sorts can cause the death of victims.

Exposure of children to violence has great relevance in public health because it poses a risk to their full development, with a wide impact on adult life, hence it is necessary to study and compare the epidemiological profiles of affected populations. In Brazil, epidemiological studies of ACE are still scarce, and different evaluation instruments or non-standard questionnaires have been used, which hinders comparability between results and the contrast with international studies. Thus, considering that there is an instrument proposed by the WHO to establish world parameters, it is pertinent to undertake a cross-cultural adaptation of ACE-IQ to be used with the Brazilian population, since the creation and development of new evaluation instruments is a complex process involving several steps to demonstrate satisfactory psychometric properties^29^.

Cross-cultural adaptation is a thorough procedure that analyzes the use of language considering the cultural context and lifestyle of the population to which the instrument is intended. It is necessary to ensure that the original instrument and the one adapted to the new language are equivalent conceptually, semantically, in operation, measurement and items^30,31^.

To provide a standardized ACE evaluation instrument to be used in Brazil, thereby increasing comparability with other studies and contributing to the validation of the new WHO-proposed instrument, a cross-cultural adaptation of the ACE-IQ was undertaken. This paper presents the semantic equivalence of the Portuguese version of the ACE-IQ, named *Questionário Internacional de Experiências Adversas na Infância* (EAI-QI).

## METHODS

The cross-cultural adaptation process (Figure 1) used in this study was based on the methodology used by Reichenheim and Moraes (2007)^32^ and the recommendations for adaptation of the ACE-IQ available on the WHO website^33,34^. The ACE-IQ was designed to be used both in self-applied format and face-to-face interviews^27,28^. For the cross-cultural adaptation process, the questionnaire was used in the interview form.

**Figure:**
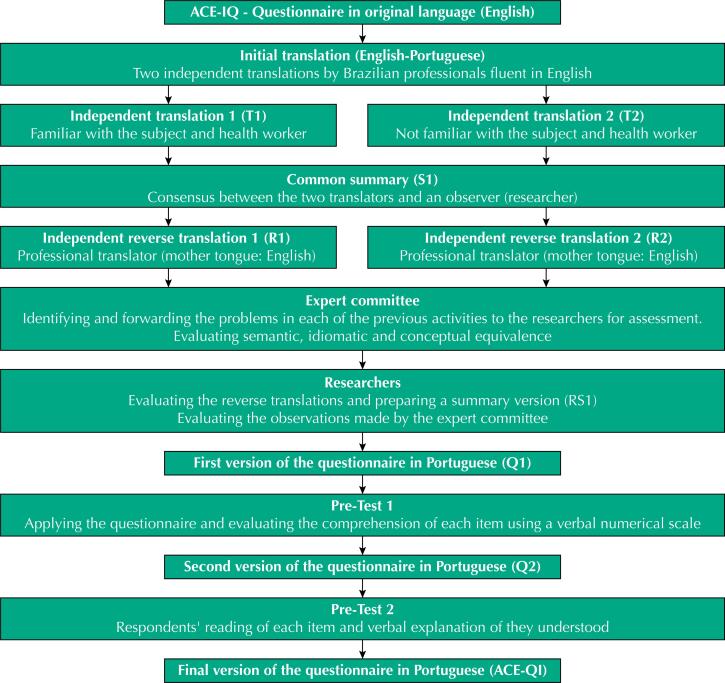
Flowchart of the process of cross-cultural adaptation of Adverse Childhood Experiences International Questionnaire (ACE-IQ).

The cross-cultural adaptation process was divided into two phases. The first phase consisted in assessing conceptual and item equivalence. This was done by the researchers and the result was an integrative review of the most used instruments over the last 10 years^26^. Initially, informal thematic discussions were held at the Center for Studies and Research in Psychiatric Epidemiology at Universidade Federal do Espírito Santo (CEPEP/UFES). The discussions were coordinated by MCV and included masters and PhDs in public health. The goal was to discuss and verify the relevance of the items of the original instrument to collect information on ACE in Brazil's context. The discussions were based on the main questionnaires and inquiries conducted in international and local context, identified by a systematic review study of publications available until 2003^24^.

Then, the second phase of cross-cultural adaptation started, consisting in an assessment of semantic equivalence. This phase was subdivided into five stages. Initially (Stage 1), two translations (T1 and T2) of the original instrument were made from English into Portuguese by two independent translators with Portuguese as their mother tongue. One of the translators is medically trained and has experience in the area of violence against women, while the other is trained in nutrition science with professional experience in chronic non-communicable diseases. Still in Step 1, the researchers located the discrepancies in the translations and asked the translators to reach a consensus in order to achieve a summarized version (S1) of the questionnaire in Portuguese (Step 1).

In the second stage (Stage 2), S1 underwent two reverse translations (to English) by independent translators with English as their mother tongue, one by a specialized company (R1) and the other by an English language teacher (R2).

To conduct the third stage (Stage 3), 13 professionals graduated in different areas were invited to compose the expert committee. The invitation was sent by e-mail with a letter explaining the role of the committee, besides brief background information about the ACE-IQ questionnaire and the methods used in the cross-cultural adaptation of instruments. Eight professionals agreed to participate in Stage 3, six of whom had a medical degree, one a psychology degree and one an arts degree specialized in anthropology. The other seven were PhDs in different areas (epidemiology/collective health, physiology, psychiatry, psychopharmacology). The composition of an expert committee on different areas aims to attest to the broad understanding of the questionnaire, considering its applicability for evaluating the general population^35^.

The expert committee was assigned the evaluation of semantic, conceptual and idiomatic equivalence between the original instrument and the two reverse translations in order to verify the literal correspondence of each translated item and evaluate the correspondence with the original meaning, considering the meaning that some words take in the new cultural context where the instrument will be used. The purpose of this process is finding possible errors made in the previous stages and collecting suggestions to better adapt a first version of the questionnaire (Q1) into Portuguese. All committee members had access to the versions produced in each stage (S1, R1 and R2), as well as the original English language questionnaire, for comparison.

The experts were asked to evaluate, for each item and for the questionnaire as a whole, whether the following items were adequate: correspondence of S1 with the original version, relevance/importance, clarity, vocabulary, objectivity, applicability to Brazil's context and instructional sequence of the items. To evaluate these items, a 1 to 4 Likert scale was used (1 = adequate; 2 = adequate with few changes; 3 = adequate with many changes and 4 = inadequate). By analyzing the evaluation of the items made by the expert committee, the content validity index (CVI) was estimated in an adapted manner [where CVI = (number of answers 1 and 2)/(total number of experts)]. A value above 0.8 was considered to be the adequate index 0.8^35^. The experts were also asked to evaluate the correspondence of the reverse translations (R1 and R2) of each item in relation to the original questionnaire (1 = unchanged; 2 = few changes; 3 = many changes and 4 = completely changed)^35,36^.

The fourth stage (Stage 4) consisted in producing a summarized version of the reverse translations (RS1) and the Q1 version. The summarized version resulted from the comparison of the two reverse translations evaluated by the expert committee, by a third translator (R3), proficient and fluent in both languages, with medical training and extensive knowledge about the subject of adverse experiences in childhood, as well as a collective health worker (MCV). The Q1 version was then produced considering the evaluation by the expert committee of the adequacy of each item of the questionnaire and their proposals for better adequacy in items considered inadequate, provided that they were relevant to the original context of ACE-IQ by the researchers, according to WHO instructional documents^34^.

The last stage (Step 5) of semantic equivalence was running a pre-test (Pre-Test 1) with the Q1 version in a sample of student volunteers and employees of a graduate program, consisting of nine women and two men, to evaluate acceptability and understanding of the items and the emotional reaction of the respondents to the topics addressed^32^. The age of the participants ranged from 28 to 48 years, and schooling ranged from high school to doctorate. The questionnaire (Q1) was applied in a standardized manner, in face-to-face interviews conducted by one of the researchers (FGP).

The interviewees used an adapted^25^ numerical verbal scale to evaluate how easy it was to understand each item of the questionnaire. The guiding question for all items was “Did you understand what was asked?”, with three possible answers, as follows: 1 = I did not understand anything; 2 = I understood, but I have questions; 3 = I understood perfectly and have no questions. Respondents were also asked to explain what they understood about each item of the questionnaire in order to assess whether each question replicated the meaning of the original item. At the end of the questionnaire, the participants answered the following questions, still in a face-to-face interview with the researcher (FGP): a) do all the items of the instrument have satisfactory answer options?; b) are you familiar with the terms used?; c) do you find any terms or passages ambiguous or difficult to understand?; and d) regarding the items you did not understand, or in a general way, would you like make any suggestions for improving the questionnaire? The questions were open-ended, and all the answers were explored and noted.

The comments and questions collected in this stage were considered in the making of a second version (Q2), presented to ten new volunteers, eight women and two men. This group was composed of students and employees aged between 19 and 42 years from a public health graduate program, with education ranging from the technical high school to doctorate. The participants read the questionnaire aloud in face-to-face interviews and explained what they understood about each item (pre-test 2). The Q2 version was then adjusted and the final version of the ACE-IQ, adapted for use in Brazil, was produced. It was named “Questionário Internacional de Experiências Adversas na Infância (EAI-QI)”, available in Chart. The numbering and coding of all items was kept as in the original instrument to facilitate the comparison with other studies and the compilation of multiple databases (Chart).

### Ethical Procedures

Authorization to undertake the process of cross-cultural adaptation of the ACE-IQ was requested to the WHO violence prevention coordinator, and granted via e-mail.

The ACE-IQ cross-cultural adaptation project was approved by the Research Ethics Committee of the UFES Health Sciences Center (CEPE/UFES 2,141,917). All participants signed the informed consent form.

**Box:** Original version of the *Adverse Childhood Experiences International Questionnaire* (ACE-IQ) and versions produced in Portuguese and adapted for use in Brazil.

	ORIGINAL VERSION	FIRST VERSION (Q1) Applied in Pre-Test 1	FINAL VERSION OF THE QUESTIONNAIRE Considering the results of Pre-Test 2
	Adverse Childhood Experiences International Questionnaire (ACE-IQ)	International Adverse Childhood Experiences Questionnaire (ACE-IQ)	International Adverse Childhood Experiences Questionnaire (ACE-IQ)
0	**DEMOGRAPHIC INFORMATION**	**DEMOGRAPHIC INFORMATION**	**DEMOGRAPHIC INFORMATION**
0.1 [C1]	Sex (*Record Male/Female as observed*) [ ] Male [ ] Female	*Sexo (Marque de acordo com o observado)* *[ ] Masculino* *[ ] Feminino*	*Sexo (Marque de acordo com o observado)* *[ ] Masculino* *[ ] Feminino*
0.2 [C2]	What is your date of birth? Day [ ][ ] Month [ ][ ] Year [ ][ ][ ][ ] Unknown *(Go to Q.C3)*	*Qual a data do seu nascimento?* *Dia [ ] mês [ ] ano [ ][ ][ ][ ]* *Não sabe/não informou [ ] **(Vá para Q.C3)***	*Qual a data do seu nascimento?* *Dia [ ] mês [ ] ano [ ][ ][ ][ ]* *Não sabe (não sei)/não informou (não quero informar) [ ] **(Vá para Q.C3**)*
0.3 [C3]	How old are you? [ ] [ ]	*Qual é a sua idade?* *[ ] [ ]*	*Qual é a sua idade?* *[ ] [ ]*
0.4 [C.4]	What is your [*insert relevant ethnic group/racial group/cultural group/others*] background? [*Locally defined*] [*Locally defined*] [*Locally defined*] Refused	*Qual das seguintes alternativas você considera como a sua raça/cor de pele?* *[ ] Branca* *[ ] Preta* *[ ] Parda* *[ ] Amarela* *[ ] Indígena* *[ ] Outras: _____________________________* *[ ] Não quis responder*	*Qual das seguintes alternativas você considera como a sua raça/cor da pele?* *[ ] Branca* *[ ] Preta* *[ ] Parda* *[ ] Amarela* *[ ] Indígena* *[ ] Outras: _____________________________* *[ ] Não declarada/Não quero declarar* *[ ] Não sabe informar/Não sei informar*
0.5 [C5]	What is the highest level of education you have completed? No formal schooling Less than primary school Primary school completed Secondary/High school completed College/University completed Post graduate degree Refused	*Qual é o seu mais alto nível de escolaridade?* *[ ] Não frequentou a escola* *[ ] Ensino Fundamental I (até 5° ano ou 4ᵃ série)* *[ ] Ensino FundamentalIincompleto (até 9° ano ou 8ᵃ série)* *[ ] Ensino Fundamental completo* *[ ] Ensino Médio incompleto* *[ ] Ensino Médio completo* *[ ] Ensino Superior incompleto* *[ ] Ensino Superior completo* *[ ] Pós-Graduação* *[ ] Não quis responder*	*Qual é o seu nível de escolaridade mais alto?* *[ ] Não frequentou a escola* *[ ] Ensino Fundamental I (até 5° ano ou 4ᵃ série)* *[ ] Ensino Fundamental II incompleto (até 9° ano ou 8ᵃ série)* *[ ] Ensino Fundamental completo* *[ ] Ensino Médio incompleto* *[ ] Ensino Médio completo* *[ ] Ensino Superior incompleto* *[ ] Ensino Superior completo* *[ ] Pós-Graduação* *[ ] Não quis responder/Não quero responder*
0.6 [C6]	Which of the following best describes your main work status over the last 12 months? Government employee Non-government employee Self-employed Non-paid Student Homemaker Retired Unemployed (able to work) Unemployed (unable to work) Refused	*Quais das seguintes opções melhor descrevem a sua situação de trabalho nos últimos 12 meses?* *(assinale todas as alternativas que se aplicam)* *[ ] Funcionário público* *[ ] Empregado do setor privado* *[ ] Trabalhador autônomo/trabalha por conta própria/Empregador* *[ ] Trabalho não remunerado/trabalho voluntário* *[ ] Estudante* *[ ] Dona de casa/Trabalho doméstico não remunerado* *[ ] Aposentado(a)* *[ ] Desempregado(a) – com capacidade de trabalhar* *[ ] Desempregado(a) – sem capacidade de trabalhar* *[ ] Outros:_____________________________* *[ ] Não quis responder*	*Quais das seguintes opções melhor descrevem a sua principal situação de trabalho nos últimos 12 meses?* *(assinalar todas as alternativas que se aplicam)* *[ ] Funcionário público/Vínculo com o setor público* *[ ] Empregado do setor privado* *[ ] Trabalhador autônomo/trabalha por conta própria/Empregador* *[ ] Trabalho não remunerado/trabalho voluntário* *[ ] Estudante* *[ ] Dona de casa/Trabalho doméstico não remunerado* *[ ] Aposentado(a)* *[ ] Desempregado(a) – com capacidade de trabalhar* *[ ] Desempregado(a) – sem capacidade de trabalhar* *[ ] Outros:_____________________________* *[ ] Não quis responder/Não quero responder*
0.7 [C7]	What is your civic status? Married (Go to Q.M2) Living as couple Divorced or separated Single Widowed (Go to Q.M2) Other Refused	Qual o seu estado civil? [ ] Casado(a) ***(Vá para Q.M2)*** [ ] Vive com companheiro(a) [ ] Divorciado(a)/separado(a) [ ] Solteiro(a) [ ] Viúvo(a) ***(Vá para Q.M2)*** [ ] Outro: ______________________________ [ ] Não quis responder	*Qual o seu estado civil?* *[ ] Casado(a)* ***(Vá para Q.M2)*** *[ ] Vive com companheiro(a)* *[ ] Divorciado(a)/separado(a)* *[ ] Solteiro(a)* *[ ] Viúvo(a)* ***(Vá para Q.M2)*** *[ ] Outro: ______________________________* *[ ] Não quis responder/Não quero responder*
**1**	**MARRIAGE**	***CASAMENTO***	***CASAMENTO***
1.1 [M1]	Have you ever been married? Yes No (Go to Q.M5) Refused	*Você já foi casado(a)?* *[ ] Sim* *[ ] Não* ***(Vá para Q.M5)*** *[ ] Não quis responder*	*Você já foi casado(a)?* *[ ] Sim* *[ ] Não* ***(Vá para Q.M5)*** *[ ] Não quis responder/Não quero responder*
1.2 [M2]	At what age were you first married? Age [ ][ ] Refused	*Com que idade você se casou pela primeira vez?* *Idade [ ] [ ]* *[ ] Não quis responder*	*Com que idade você se casou pela primeira vez?* *Idade [ ] [ ]* *[ ] Não quis responder/Não quero responder*
1.3 [M3]	At the time of your first marriage did you yourself choose your husband/wife? Yes (Go to Q.M5) No Don't know/Not sure Refused	*Quando você se casou pela primeira vez, foi você mesmo(a) quem escolheu seu marido/esposa?* *[ ] Sim* ***(Vá para Q.M5)*** *[ ] Não* *[ ] Não sabe/Não tem certeza* *[ ] Não quis responder*	*Quando você se casou pela primeira vez, foi você mesmo(a) quem escolheu seu marido/esposa?* *[ ] Sim* ***(Vá para Q.M5)*** *[ ] Não* *[ ] Não sabe (Não sei)/Não tem certeza (Não tenho certeza)* *[ ] Não quis responder/Não quero responder*
1.4 [M4]	At the time of your first marriage if you did not choose your husband/wife yourself, did you give your consent to the choice? Yes No Refused	*Quando você se casou pela primeira vez, se não foi você quem escolheu seu marido/esposa, você concordou com a escolha?* *[ ] Sim* *[ ] Não* *[ ] Não quis responder*	*Quando você se casou pela primeira vez, se não foi você quem escolheu seu marido/esposa, você concordou com a escolha?* *[ ] Sim* *[ ] Não* *[ ] Não quis responder/Não quero responder*
1.5 [M5]	If you are a mother or father what was your age when your first child was born? Age [ ][ ] Not applicable Refused	*Se você já teve filhos, qual era sua idade quando nasceu seu(sua) primeiro(a) filho(a)?* *Idade [ ] [ ]* *Não se aplica [ ]* *[ ] Não quis responder*	*Se você já teve filhos, qual era sua idade quando nasceu seu(sua) primeiro(a) filho(a)?* *Idade [ ] [ ]* *Não se aplica [ ]* *[ ] Não quis responder/Não quero responder*
**2**	**RELATIONSHIP WITH PARENTS/GUARDIANS** When you were growing up, during the first 18 years of your life…	***RELACIONAMENTO COM OS PAIS OU RESPONSÁVEIS*** *Durante o período de crescimento, nos primeiros 18 anos de sua vida…*	***RELACIONAMENTO COM OS PAIS OU RESPONSÁVEIS*** *A partir de agora, todas as perguntas estão relacionadas ao período em que você estava crescendo, até os seus 18 anos.*
2.1 [P1]	Did your parents/guardians understand your problems and worries? Always Most of the time Sometimes Rarely Never Refused	*Seus pais/responsáveis compreendiam seus problemas e preocupações?* *[ ] Sempre* *[ ] Na maioria das vezes* *[ ] Às vezes* *[ ] Raramente* *[ ] Nunca* *[ ] Não quis responder*	*Seus pais/responsáveis compreendiam seus problemas e preocupações?* *[ ] Sempre* *[ ] Na maioria das vezes* *[ ] Às vezes* *[ ] Raramente* *[ ] Nunca* *[ ] Não quis responder/Não quero responder*
2.2 [P2]	Did your parents/guardians **really** know what you were doing with your free time when you were not at school or work? Always Most of the time Sometimes Rarely Never Refused	*Seus pais/responsáveis **realmente** sabiam o que você estava fazendo no seu tempo livre, quando não estava na escola ou no trabalho?* *[ ] Sempre* *[ ] Na maioria das vezes* *[ ] Às vezes* *[ ] Raramente* *[ ] Nunca* *[ ] Não quis responder*	*Seus pais/responsáveis **realmente** sabiam o que você estava fazendo no seu tempo livre, quando não estava na escola ou no trabalho?* *[ ] Sempre* *[ ] Na maioria das vezes* *[ ] Às vezes* *[ ] Raramente* *[ ] Nunca* *[ ] Não quis responder/Não quero responder*
3			
3.1 [P3]	How often did your parents/guardians **not** give you enough food even when they could easily have done so? Many times A few times Once Never Refused	*Com que frequência seus pais/responsáveis **não** lhe davam comida suficiente, mesmo que pudessem facilmente fazer isso?* *[ ] Muitas vezes* *[ ] Poucas vezes* *[ ] Uma vez* *[ ] Nunca* *[ ] Não quis responder*	*Com que frequência seus pais/responsáveis **não** lhe davam comida suficiente, mesmo que pudessem facilmente oferecer um alimento para você?* *[ ] Muitas vezes* *[ ] Poucas vezes* *[ ] Uma vez* *[ ] Nunca* *[ ] Não quis responder/Não quero responder*
3.2 [P4]	Were your parents/guardians too drunk or intoxicated by drugs to take care of you? Many times A few times Once Never Refused	*Seus pais/responsáveis ficavam muito embriagados ou sob o efeito de drogas quando cuidavam de você?* *[ ] Muitas vezes* *[ ] Poucas vezes* *[ ] Uma vez* *[ ] Nunca* *[ ] Não quis responder*	*Seus pais/responsáveis ficavam muito embriagados ou sob o efeito de drogas quando cuidavam de você?* *[ ] Muitas vezes* *[ ] Poucas vezes* *[ ] Uma vez* *[ ] Nunca* *[ ] Não quis responder/Não quero responder*
3.3 [P5]	How often did your parents/guardians **not** send you to school even when it was available? Many times A few times Once Never Refused	*Com que frequência seus pais/responsáveis **não** lhe mandavam para a escola, mesmo que devessem fazer isso?* *[ ] Muitas vezes* *[ ] Poucas vezes* *[ ] Uma vez* *[ ] Nunca* *[ ] Não quis responder*	*Com que frequência seus pais/responsáveis **não** lhe mandavam para a escola, mesmo que tivessem a obrigação de fazer isso?* *[ ] Muitas vezes* *[ ] Poucas vezes* *[ ] Uma vez* *[ ] Nunca* *[ ] Não quis responder/Não quero responder*
4	**FAMILY ENVIRONMENT** When you were growing up, during the first 18 years of your life…	***AMBIENTE FAMILIAR*** *Durante o período de crescimento, nos primeiros 18 anos de sua vida…*	***AMBIENTE FAMILIAR*** *Nos primeiros 18 anos da sua vida…*
4.1 [F1]	Did you live with a household member who was a problem drinker or alcoholic, or misused street or prescription drugs? Yes No Refused	*Você morou com alguém que tinha problemas com álcool ou era alcoólatra, ou que abusava de drogas ilícitas ou de medicamentos controlados?* *[ ] Sim* *[ ] Não* *[ ] Não quis responder*	*Você morou com alguém que tinha problemas com álcool ou era alcoólatra, ou que abusava de drogas ilícitas ou de medicamentos controlados?* *[ ] Sim* *[ ] Não* *[ ] Não quis responder/Não quero responder*
4.2 [F2]	Did you live with a household member who was depressed, mentally ill or suicidal? Yes No Refused	*Você morou com alguém que estava deprimido, ou tinha alguma doença mental ou intenção suicida?* *[ ] Sim* *[ ] Não* *[ ] Não quis responder*	*Você morou com alguém que estava deprimido, ou tinha alguma doença mental ou intenção suicida?* *[ ] Sim* *[ ] Não* *[ ] Não quis responder/Não quero responder*
4.3 [F3]	Did you live with a household member who was ever sent to jail or prison? Yes No Refused	*Você morou com alguém que alguma vez tenha sido levado pra cadeia ou mandado pra prisão?* *[ ] Sim* *[ ] Não* *[ ] Não quis responder*	*Você morou com alguém que alguma vez tenha sido levado pra cadeia ou mandado pra prisão?* *[ ] Sim* *[ ] Não* *[ ] Não quis responder/Não quero responder*
4.4 [F4]	Were your parents ever separated or divorced? Yes No Not applicable Refused	*Nesse período, seus pais alguma vez se separaram ou se divorciaram?* *[ ] Sim* *[ ] Não* *[ ] Não se aplica* *[ ] Não quis responder*	*Nesse período (até os seus 18 anos) seus pais alguma vez se separaram ou se divorciaram?* *[ ] Sim* *[ ] Não* *[ ] Não se aplica* *[ ] Não quis responder/Não quero responder*
4.5 [F5]	Did your mother, father or guardian die? Yes No Don't know/Not sure Refused	*Sua mãe, pai ou responsável faleceram (nesse período)?* *[ ] Sim* *[ ] Não* *[ ] Não sabe/Não tem certeza* *[ ] Não quis responder*	*Sua mãe, pai ou responsável faleceram (nesse período)?* *[ ] Sim* *[ ] Não* *[ ] Não sabe/Não tem certeza* *[ ] Não quis responder/Não quero responder*
	These next questions are about certain things you may actually have heard or seen **IN YOUR HOME**. These are things that may have been done to another household member but not necessarily to you. When you were growing up, during the first 18 years of your life…	*As próximas perguntas são sobre algumas situações/coisas que você pode ter ouvido ou visto **EM SUA CASA**. São situações/coisas que podem ter acontecido com outros moradores da sua casa, não necessariamente com você.* *Durante o período de crescimento, nos primeiros 18 anos de sua vida…*	*As próximas perguntas estão relacionadas a algumas situações que você pode ter ouvido ou visto **EM SUA CASA**. São situações que podem ter acontecido **COM OS MORADORES DA SUA CASA, NÃO COM VOCÊ**.* *Nos primeiros 18 anos da sua vida…*
4.6 [F6]	Did you see or hear a parent or household member in your home being yelled at, screamed at, sworn at, insulted or humiliated? Many times A few times Once Never Refused	*Você viu ou ouviu algum de seus pais ou alguém que morava na sua casa recebendo gritos ou berros, ou sendo xingado, insultado ou humilhado?* *[ ] Muitas vezes* *[ ] Poucas vezes* *[ ] Uma vez* *[ ] Nunca* *[ ] Não quis responder*	*Você viu ou ouviu alguém que morava na sua casa recebendo gritos ou berros, ou sendo xingado, insultado ou humilhado?* *[ ] Muitas vezes* *[ ] Poucas vezes* *[ ] Uma vez* *[ ] Nunca* *[ ] Não quis responder/Não quero responder*
4.7 [F7]	Did you see or hear a parent or household member in your home being slapped, kicked, punched or beaten up? Many times A few times Once Never Refused	*Você viu ou ouviu algum de seus pais ou alguém que morava na sua casa sendo estapeado, chutado, socado ou surrado?* *[ ] Muitas vezes* *[ ] Poucas vezes* *[ ] Uma vez* *[ ] Nunca* *[ ] Não quis responder*	*Você viu ou ouviu alguém que morava na sua casa sendo estapeado, chutado, socado ou surrado?* *[ ] Muitas vezes* *[ ] Poucas vezes* *[ ] Uma vez* *[ ] Nunca* *[ ] Não quis responder/Não quero responder*
4.8 [F8]	Did you see or hear a parent or household member in your home being hit or cut with an object, such as a stick (or cane), bottle, club, knife, whip etc.? Many times A few times Once Never Refused	*Você viu ou ouviu algum de seus pais ou alguém que morava na sua casa ser atingido ou cortado com algum objeto, como uma vara (ou bengala), garrafa, porrete, faca, chicote, etc?* *[ ] Muitas vezes* *[ ] Poucas vezes* *[ ] Uma vez* *[ ] Nunca* *[ ] Não quis responder*	*Você viu ou ouviu alguém que morava na sua casa ser agredido ou cortado com algum objeto, como uma vara (ou bengala), garrafa, porrete, faca, chicote, ou algum outro objeto?* *[ ] Muitas vezes* *[ ] Poucas vezes* *[ ] Uma vez* *[ ] Nunca* *[ ] Não quis responder/Não quero responder*
5	These next questions are about certain things **YOU** may have experienced. When you were growing up, during the first 18 years of your life…	*Estas próximas perguntas são sobre certas coisas que **VOCÊ** pode ter vivenciado.* *Durante o período de crescimento, nos primeiros 18 anos de sua vida…*	*As próximas perguntas estão relacionadas a algumas situações que **VOCÊ** pode ter vivenciado.* *Nos primeiros 18 anos da sua vida…*
5.1 [A1]	Did a parent, guardian or other household member yell, scream or swear at you, insult or humiliate you? Many times A few times Once Never Refused	*Algum de seus pais ou responsáveis ou alguém que morava na sua casa gritou, berrou, xingou, insultou ou humilhou você?* *[ ] Muitas vezes* *[ ] Poucas vezes* *[ ] Uma vez* *[ ] Nunca* *[ ] Não quis responder*	*Algum de seus pais ou responsáveis ou alguém que morava na sua casa gritou, berrou, xingou, insultou ou humilhou você?* *[ ] Muitas vezes* *[ ] Poucas vezes* *[ ] Uma vez* *[ ] Nunca* *[ ] Não quis responder/Não quero responder*
5.2 [A2]	Did a parent, guardian or other household member threaten to, or actually, abandon you or throw you out of the house? Many times A few times Once Never Refused	*Algum de seus pais ou responsáveis ou alguém que morava na sua casa ameaçou abandonar ou expulsar você de casa, ou de fato fez isso?* *[ ] Muitas vezes* *[ ] Poucas vezes* *[ ] Uma vez* *[ ] Nunca* *[ ] Não quis responder*	*Algum de seus pais ou responsáveis ou alguém que morava na sua casa **ameaçou** abandonar ou expulsar você de casa, **ou de fato fez isso**?* *[ ] Muitas vezes* *[ ] Poucas vezes* *[ ] Uma vez* *[ ] Nunca* *[ ] Não quis responder/Não quero responder*
5.3 [A3]	Did a parent, guardian or other household member spank, slap, kick, punch or beat you up? Many times A few times Once Never Refused	*Algum de seus pais ou responsáveis ou alguém que morava na sua casa bateu, deu tapas, chutou, socou ou surrou você?* *[ ] Muitas vezes* *[ ] Poucas vezes* *[ ] Uma vez* *[ ] Nunca* *[ ] Não quis responder*	*Algum de seus pais ou responsáveis ou alguém que morava na sua casa bateu, chutou, socou ou surrou você?* *[ ] Muitas vezes* *[ ] Poucas vezes* *[ ] Uma vez* *[ ] Nunca* *[ ] Não quis responder/Não quero responder*
			*Qual a intensidade dessas agressões?* *[ ] Leve* *[ ] Moderada* *[ ] Intensa* *[ ] Muito intensa* *[ ] Não quis responder/Não quero responder*
5.4 [A4]	Did a parent, guardian or other household member hit or cut you with an object, such as a stick (or cane), bottle, club, knife, whip etc? Many times A few times Once Never Refused	*Algum de seus pais ou responsáveis ou alguém que morava na sua casa atingiu ou cortou com algum objeto, como uma vara (ou bengala), garrafa, porrete, faca, chicote, etc?* *[ ] Muitas vezes* *[ ] Poucas vezes* *[ ] Uma vez* *[ ] Nunca* *[ ] Não quis responder*	*Algum de seus pais ou responsáveis ou alguém que morava na sua casa agrediu ou cortou você com algum objeto, como uma vara (ou bengala), garrafa, porrete, faca, chicote, ou algum outro objeto?* *[ ] Muitas vezes* *[ ] Poucas vezes* *[ ] Uma vez* *[ ] Nunca* *[ ] Não quis responder/Não quero responder*
			*As próximas perguntas são sobre situações de assédio/molestação sexual que VOCÊ possa ter vivenciado até os 18 anos…*
5.5 [A5]	Did someone touch or fondle you in a sexual way when you did not want them to? Many times A few times Once Never Refused	*Alguém tocou ou acariciou você de uma forma sexual sem que você quisesse?* *[ ] Muitas vezes* *[ ] Poucas vezes* *[ ] Uma vez* *[ ] Nunca* *[ ] Não quis responder*	*Algum de seus pais ou responsáveis ou alguém que morava na sua casa ou algum conhecido ou desconhecido **tocou ou acariciou** você de uma forma sexual sem que você quisesse?* *[ ] Muitas vezes* *[ ] Poucas vezes* *[ ] Uma vez* *[ ] Nunca* *[ ] Não quis responder/Não quero responder*
			*Foi alguém que morava na sua casa?* *[ ] Sim* *[ ] Não* *[ ] Não quis responder/Não quero responder*
5.6 [A6]	Did someone make you touch their body in a sexual way when you did not want them to? Many times A few times Once Never Refused	*Alguém fez com que você tocasse o corpo dele(a) de uma forma sexual sem que você quisesse fazer isso?* *[ ] Muitas vezes* *[ ] Poucas vezes* *[ ] Uma vez* *[ ] Nunca* *[ ] Não quis responder*	*Algum de seus pais ou responsáveis ou alguém que morava na sua casa ou algum conhecido ou desconhecido **fez com que você tocasse** o corpo dele(a) de uma forma sexual sem que você quisesse fazer isso?* *[ ] Muitas vezes* *[ ] Poucas vezes* *[ ] Uma vez* *[ ] Nunca* *[ ] Não quis responder/Não quero responder*
			*Foi alguém que morava na sua casa?* *[ ] Sim* *[ ] Não* *[ ] Não quis responder/Não quero responder*
5.7 [A7]	Did someone attempt oral, anal, or vaginal intercourse with you when you did not want them to? Many times A few times Once Never Refused	*Alguém tentou fazer sexo oral, anal ou vaginal com você sem que você quisesse?* *[ ] Muitas vezes* *[ ] Poucas vezes* *[ ] Uma vez* *[ ] Nunca* *[ ] Não quis responder*	*Algum de seus pais ou responsáveis ou alguém que morava na sua casa ou algum conhecido ou desconhecido **tentou fazer** sexo oral, anal ou vaginal com você sem que você quisesse?* *[ ] Muitas vezes* *[ ] Poucas vezes* *[ ] Uma vez* *[ ] Nunca* *[ ] Não quis responder/Não quero responder*
			*Foi alguém que morava na sua casa?* *[ ] Sim* *[ ] Não* *[ ] Não quis responder/Não quero responder*
5.8 [A8]	Did someone actually have oral, anal, or vaginal intercourse with you when you did not want them to? Many times A few times Once Never Refused	*Alguém já fez sexo oral, anal ou vaginal com você sem que você quisesse?* *[ ] Muitas vezes* *[ ] Poucas vezes* *[ ] Uma vez* *[ ] Nunca* *[ ] Não quis responder*	*Algum de seus pais ou responsáveis ou alguém que morava na sua casa ou algum conhecido ou desconhecido **já fez** sexo oral, anal ou vaginal com você sem que você quisesse?* *[ ] Muitas vezes* *[ ] Poucas vezes* *[ ] Uma vez* *[ ] Nunca* *[ ] Não quis responder/Não quero responder*
			*Foi alguém que morava na sua casa?* *[ ] Sim* *[ ] Não* *[ ] Não quis responder/Não quero responder*
**6**	**PEER VIOLENCE**	***VIOLÊNCIA ENTRE PARES***	
	These next questions are about BEING BULLIED when you were growing up. Bullying is when a young person or group of young people say or do bad and unpleasant things to another young person. It is also bullying when a young person is teased a lot in an unpleasant way or when a young pers on is left out of things on purpose. It is not bullying when two young people of about the same strength or power argue or fight or when teasing is done in a friendly and fun way. When you were growing up, during the first 18 years of your life…	*As próximas perguntas são sobre **SOFRER BULLYING** quando você estava crescendo.* *O bullying ocorre quando um jovem (criança ou adolescente) ou um grupo de jovens diz ou faz coisas ruins ou desagradáveis para outro jovem (criança ou adolescente).* *Também é* bullying *quando uma pessoa jovem é provocada de forma desagradável ou quando é deixada de fora das atividades de propósito. Não é* bullying *quando dois jovens com a mesma força ou poder discutem ou brigam ou quando a provocação ocorre de forma amistosa e divertida.* *Durante o período de crescimento, nos primeiros 18 anos de sua vida…*	*As próximas perguntas estão relacionadas ao* **BULLYING**. *O* bullying *ocorre quando um jovem ou um grupo de jovens diz ou faz coisas ruins ou desagradáveis para outro jovem.* *Também é* bullying *quando uma pessoa jovem é provocada de forma desagradável ou quando é deixada de fora das atividades de propósito. Não é* bullying *quando dois jovens com a mesma força ou poder discutem ou brigam ou quando a provocação ocorre de forma amistosa e divertida.* *Nos primeiros 18 anos da sua vida…*
6.1 [V1]	How often were you bullied? Many times A few times Once Never (Go to Q.V3) Refused	*Com que frequência você sofria* bullying*?* *[ ] Muitas vezes* *[ ] Poucas vezes* *[ ] Uma vez* *[ ] Nunca* (***Vá para Q.V3)*** *[ ] Não quis responder*	*Com que frequência você sofria* bullying? *[ ] Muitas vezes* *[ ] Poucas vezes* *[ ] Uma vez* *[ ] Nunca* (***Vá para Q.V3***) *[ ] Não quis responder/Não quero responder*
6.2 [V2]	How were you bullied most often? I was hit, kicked, pushed, shoved around, or locked indoors I was made fun of because of my race, nationality or colour I was made fun of because of my religion I was made fun of with sexual jokes, comments, or gestures I was left out of activities on purpose or completely ignored I was made fun of because of how my body or face looked I was bullied in some other way Refused	*Qual era a forma mais frequente de* bullying *que você sofria?* *[ ] Me batiam, esbarravam em mim, era chutado(a), empurrado(a), ou trancado(a) em lugares fechados* *[ ] Era zoado(a) (Riam de mim/me zoavam) por causa da minha raça, nacionalidade ou cor de pele* *[ ] Era zoado(a) (Riam de mim/me zoavam) por causa da minha religião* *[ ] Era zoado(a) (Riam de mim/me zoavam) por meio de brincadeiras ou comentários de cunho sexual, ou gestos obcenos* *[ ] Era excluído(a) de atividades de propósito ou completamente ignorado(a)* *[ ] Era zoado(a) (Riam de mim/me zoavam) por causa da aparência do meu corpo ou do meu rosto* *[ ] Eu sofria alguma outra forma de* bullying *[ ] Não quis responder*	*Qual era a forma **mais frequente** de* bullying *que você sofria?* ***(Assinalar apenas uma opção)*** *[ ] Me batiam, esbarravam em mim, era chutado(a), empurrado(a), ou trancado(a) em lugares fechados* *[ ] Era zoado(a) (debochavam de mim) por causa da minha raça, nacionalidade ou cor da pele* *[ ] Era zoado(a) (debochavam de mim) por causa da minha religião* *[ ] Era zoado(a) (debochavam de mim) por meio de brincadeiras ou comentários de cunho sexual, ou gestos obcenos* *[ ] Era excluído(a) de atividades de propósito ou completamente ignorado(a)* *[ ] Era zoado(a) (debochavam de mim) por causa da aparência do meu corpo ou do meu rosto* *[ ] Eu sofria alguma outra forma de* bullying *[ ] Não quis responder/Não quero responder*
	This next question is about **PHYSICAL FIGHTS**. A physical fight occurs when two young people of about the same strength or power choose to fight each other. When you were growing up, during the first 18 years of your life…	*A próxima pergunta é sobre **BRIGAS FÍSICAS**. Uma briga física ocorre quando dois jovens com aproximadamente a mesma força ou poder escolhem brigar um contra o outro.* *Durante o período de crescimento, nos primeiros 18 anos de sua vida…*	*A próxima pergunta é sobre **BRIGAS FÍSICAS**. Uma briga física ocorre quando dois jovens com aproximadamente a mesma força ou poder escolhem brigar um contra o outro.* *Durante o período de crescimento, nos primeiros 18 anos da sua vida…*
6.3 [V3]	Did you see or hear someone being beaten up in real life? Many times A few times Once Never Refused	*Com que frequência você se envolvia em uma briga física?* *[ ] Muitas vezes* *[ ] Poucas vezes* *[ ] Uma vez* *[ ] Nunca* *[ ] Não quis responder*	*Com que frequência você se envolvia em uma briga física?* *[ ] Muitas vezes* *[ ] Poucas vezes* *[ ] Uma vez* *[ ] Nunca* *[ ] Não quis responder/Não quero responder*
7	**WITNESSING COMMUNITY VIOLENCE** These next questions are about how often, when you were a child, **YOU** may have seen or heard certain things in your **NEIGHBOURHOOD OR COMMUNITY** (not in your home or on TV, movies, or the radio). When you were growing up, during the first 18 years of your life…	***VIOLÊNCIA NA COMUNIDADE*** *As próximas perguntas são sobre a frequência com que **VOCÊ** viu ou ouviu certas coisas na sua **VIZINHANÇA OU COMUNIDADE** quando você era criança (não na sua casa ou na TV, rádio ou em filmes).* *Durante o período de crescimento, nos primeiros 18 anos de sua vida…*	***VIOLÊNCIA NA COMUNIDADE*** *As próximas perguntas são sobre a frequência com que **VOCÊ** viu ou ouviu certas coisas na sua **VIZINHANÇA OU COMUNIDADE** (não na sua casa ou na TV, rádio ou em filmes).* *Nos primeiros 18 anos da sua vida…*
7.1 [V4]	Did you see or hear someone being beaten up in real life? Many times A few times Once Never Refused	*Você viu ou ouviu alguém sendo espancado (na vida real)?* *[ ] Muitas vezes* *[ ] Poucas vezes* *[ ] Uma vez* *[ ] Nunca* *[ ] Não quis responder*	*Você viu ou ouviu alguém sendo espancado (não na sua casa ou na TV, rádio ou em filmes)?* *[ ] Muitas vezes* *[ ] Poucas vezes* *[ ] Uma vez* *[ ] Nunca* *[ ] Não quis responder/Não quero responder*
7.2 [V5]	Did you see or hear someone being stabbed or shot in real life? Many times A few times Once Never Refused	*Você viu ou ouviu alguém sendo esfaqueado ou levando um tiro (na vida real)?* *[ ] Muitas vezes* *[ ] Poucas vezes* *[ ] Uma vez* *[ ] Nunca* *[ ] Não quis responder*	*Você viu ou ouviu alguém sendo esfaqueado ou levando um tiro (não na sua casa ou na TV, rádio ou em filmes)?* *[ ] Muitas vezes* *[ ] Poucas vezes* *[ ] Uma vez* *[ ] Nunca* *[ ] Não quis responder/Não quero responder*
7.3 [V6]	Did you see or hear someone being threatened with a knife or gun in real life? Many times A few times Once Never Refused	*Você viu ou ouviu alguém ser ameaçado(a) com uma faca ou arma de fogo (na vida real)?* *[ ] Muitas vezes* *[ ] Poucas vezes* *[ ] Uma vez* *[ ] Nunca* *[ ] Não quis responder*	*Você viu ou ouviu alguém ser ameaçado(a) com uma faca ou arma de fogo (não na sua casa ou na TV, rádio ou em filmes)?* *[ ] Muitas vezes* *[ ] Poucas vezes* *[ ] Uma vez* *[ ] Nunca* *[ ] Não quis responder/Não quero responder*
**8**	**EXPOSURE TO WAR/COLLECTIVE VIOLENCE**	***EXPOSIÇÃO A GUERRA/VIOLÊNCIA COLETIVA***	***VIOLÊNCIA COLETIVA***
	These questions are about whether **YOU** did or did not experience any of the following events when you were a child. The events are all to do with collective violence, including wars, terrorism, political or ethnic conflicts, genocide, repression, disappearances, torture and organized violent crime such as banditry and gang warfare. When you were growing up, during the first 18 years of your life…	*As próximas perguntas são sobre se **VOCÊ** vivenciou ou não alguma dos seguintes acontecimentos quando você era criança. Todos eles estão relacionados com violência coletiva, incluindo guerras, terrorismo, conflitos políticos ou étnicos, genocídio, repressão, desaparecimentos, tortura e crime organizado violento, como bandidagem e guerra de gangues.* *Durante o período de crescimento, nos primeiros 18 anos de sua vida…*	*As próximas perguntas são sobre se **VOCÊ** vivenciou ou não algum dos seguintes acontecimentos quando você era criança. Todos eles estão relacionados com violência coletiva, incluindo guerras ou tiroteios, terrorismo, conflitos políticos ou étnicos, genocídio, repressão ou toque de recolher, desaparecimentos, tortura e crime organizado violento, como bandidagem, tráfico de drogas e guerra de gangues.* *Nos primeiros 18 anos da sua vida…*
8.1 [V7]	Were you forced to go and live in another place due to any of these events? Many times A few times Once Never Refused	*Você foi forçado a ir viver em outro lugar devido a algum desses acontecimentos?* *[ ] Muitas vezes* *[ ] Poucas vezes* *[ ] Uma vez* *[ ] Nunca* *[ ] Não quis responder*	*Você foi forçado a ir viver em outro lugar devido a algum desses acontecimentos?* *[ ] Muitas vezes* *[ ] Poucas vezes* *[ ] Uma vez* *[ ] Nunca* *[ ] Não quis responder/Não quero responder*
8.2 [V8]	Did you experience the deliberate destruction of your home due to any of these events? Many times A few times Once Never Refused	*Você vivenciou a destruição proposital da sua casa devido a algum desses eventos?* *[ ] Muitas vezes* *[ ] Poucas vezes* *[ ] Uma vez* *[ ] Nunca* *[ ] Não quis responder*	*Você vivenciou a destruição proposital da sua casa devido a algum desses eventos?* *[ ] Muitas vezes* *[ ] Poucas vezes* *[ ] Uma vez* *[ ] Nunca* *[ ] Não quis responder/Não quero responder*
8.3 [V9]	Were you beaten up by soldiers, police, militia, or gangs? Many times A few times Once Never Refused	*Você foi espancado por soldados, policiais, milicianos ou gangues?* *[ ] Muitas vezes* *[ ] Poucas vezes* *[ ] Uma vez* *[ ] Nunca* *[ ] Não quis responder*	*Você foi espancado por soldados, policiais, milicianos, gangues ou traficantes de drogas?* *[ ] Muitas vezes* *[ ] Poucas vezes* *[ ] Uma vez* *[ ] Nunca* *[ ] Não quis responder/Não quero responder*
8.4 [V10]	Was a family member or friend killed or beaten up by soldiers, police, militia, or gangs? Many times A few times Once Never Refused	*Algum familiar ou amigo(a) foi morto ou espancado por soldados, policiais, milicianos ou gangues?* *[ ] Muitas vezes* *[ ] Poucas vezes* *[ ] Uma vez* *[ ] Nunca* *[ ] Não quis responder*	*Algum familiar ou amigo(a) foi morto ou espancado por soldados, policiais, milicianos, gangues ou traficantes de drogas?* *[ ] Muitas vezes* *[ ] Poucas vezes* *[ ] Uma vez* *[ ] Nunca* *[ ] Não quis responder/Não quero responde*r
			*Para terminar, gostaríamos de saber se até os seus 18 anos, você vivenciou algum outro evento traumático que não foi mencionado durante esta entrevista?* *[ ] Não* *[ ] Sim* *Qual(is)?* *__________________________________________* *__________________________________________* *__________________________________________*

## RESULTS

The first and last versions of the questionnaire are shown in Table with the original English language version of the instrument, in the first column. The final version of the ACE-IQ is available on the WHO website: https://www.who.int/publications/m/item/adverse-childhood-experiences-international-questionnaire-(ACe-iQ)

**Table:** Questionnaire items that presented criteria with CVI below 0.7 in the evaluation by the expert committee.

Evaluated criteria	Item C4	Item A6	% CVI > 0.8^a^
	CVI	CVI	
Correspondence with the original version	0.62	0.62	95
Relevance/importance	0.75	0.87	93
Clarity	0.37	0.62	93
Vocabulary	0.50	0.87	93
Objetividade	0.62	0.87	93
Applicable to the Brazilian model	0.50	1	95
Instructional sequence	0.75	1	98

CVI: content validity index.

a Percentage of all CVI estimations in the 43 items of the questionnaire separated by criterion evaluated by the expert committee.

Only three of the 43 items of both reverse translations, when compared by the expert committee to the original instrument, were considered to have “many changes” or “completely changed”. The discrepancies found occurred in the categories of self-declared race/color (item C4), education (C5) and occupation (C6), and were resolved by R3, according to the terms standardized by the Brazilian Institute of Geography and Statistics (IBGE). These differences had already been identified in the two initial translations. Still regarding the reverse translations, the other items were considered by the experts as “unchanged” or with “few changes”, and R2 was considered more adequate in relation to R1 regarding correspondence to the original version. All items were reviewed and, when necessary, adjusted by R3.

In the overall evaluation of the questionnaire, the CVI corresponded to 1.0 in almost all the criteria judged by the expert committee, considering the correspondence of S1 with the original version in relation to relevance/importance, clarity, vocabulary, objectivity, applicability to Brazil's context and instructional sequence of the items. Of the 43 items, only two (C4 and A6) had CVI < 0.7 (Table), requiring adjustment by R3 in both RS1 and Q1. In item C4, the experts recommended the inclusion of the answer option “others”, considering that some people may not identify with the alternatives presented. In item A6, referring to sexual abuse, the experts showed several formats for rewriting the item to emphasize the idea that the respondent was “forced” to something they did not want; however, when the original question was submitted to the pre-test, there were no interpretation difficulties; therefore, the suggestions for modification made by the expert committee were not put into practice. Generally, 73% of the evaluated criteria received the maximum score in the CVI (1.0); in 21% the CVI was 0.87; and in 4%, 0.75. Values > 0.8^33^ were considered adequate. Despite this satisfactory score, all the suggestions made by the experts were considered and, when possible and relevant, incorporated into Q1.

### Pre-Test 1

In the first pre-test, conducted with 11 participants, items P3 and V2 had the lowest comprehension averages (2.72), evaluated by the three-point verbal scale.

Item P3, which evaluates the non-provision of an adequate diet, was mistakenly interpreted by some respondents as being due to parents or guardians being unable to afford it, but physical neglect involves the non-provision of basic conditions for child development (food, clothing, medical care) when there is no economic adversity^37^. A similar interpretation occurred in item P5 (mean 2.82), in which, besides financial struggling, the impossibility of accessing, or local unavailability of, schools was suggested.

Item V2 was resolved by adding instructions for choosing only one alternative concerning the most frequent form of bullying, as the respondents tended to check more than one answer.

Item A3 was discussed among the researchers after the participants in pre-Test 1 pointed out that the expression “slapped” (among all other forms of aggression) might not mean serious physical aggression or, still, that such situations described in the item could be understood as a form of disciplinary correction instead of physical abuse. This result led to consideration and recommendations suggested by the researchers (see “Limitations of the instrument and recommendations”).

In items A5 to A8, all respondents who suffered sexual abuse expressed confusion about what was being evaluated, that is, whether the abuse was restricted to the home environment and, consequently, was committed by family member(s), or whether it included any and all sexual abuse suffered. This was because in this section, there was an abrupt and unexplained change in the way the questions were designed, compared to the previous sections. These were preceded by the phrase “[Has] any of your parents or guardians or someone who lived at your house…?”, and, in the section on sexual abuse, the introductory phrase became “[Has] anyone…”, without any specification as to the offender. According to the guidelines for using the ACE-IQ^34^, these items evaluate child sexual abuse committed by any person – family members, acquaintances or strangers to the child. To solve this difficulty and clarify the possible questions, the researchers decided to change the introductory question so that the four questions in this section were preceded by the phrase “[Has] any of your parents or guardians, or someone who lived at your house, or anyone known or unknown…”. A sub-item was also included in each question to distinguish whether or not the abusers were residents of the house (see “Limitations of the instrument and recommendations”).

The participants in Pre-Test 1 made some suggestions: 1) changing the order of item C5 from *“Qual é o seu mais alto nível de escolaridade?”* to *“Qual o seu nível de escolaridade mais alto?”*; (2) having only one choice for work situation in the last 12 months (C6), even though they found it difficult to choose only one answer; and 3) adjusting the instructions before domestic violence section (6-8), removing the word “things”, and the excerpt “not necessarily to you”; (4) replacing the word “hit” with “attacked” in the F8 key; and 5) removing the phrase “exposure to war” to include the terms “shootings and drug trafficking” in the instructions preceding the questions about collective violence (7–10).

A final item was also included in the questionnaire – “Throughout your growth period, in the first 18 years of your life, did you experience any other traumatic events that were not mentioned in the interview?” – if so, a blank space to answer (see “Limitations of the instrument and recommendations”).

### Pre-Test 2

All the adapted items and the changes suggested in the results of Pre-Test 1 aimed at using colloquial terms used in Brazil's context, which are easily understood by the general population, thus giving rise to a modified version of the questionnaire (Q2), which was evaluated in Pre-Test 2. Based on the respondents’ reading and comprehension of each item, modifications to the text were requested when the contents differed from the original version. They also suggested modifications when they thought that the question could be better formulated.

After Pre-Test 2, the researchers decided to modify part of the P3 items (“… even if they could easily offer you some food?”) and P5 (“… even if they had an obligation to do it?”) so that the question could be more easily understood (Table).

Item C6 was tested in two formats: 1) allowing the choice of all the options describing the respondent's work situation in the last 12 months; and 2) allowing only one answer describing the respondent's main work/occupation. Although the instructions in the original questionnaire require only one answer, the researchers opted to use multiple choices considering the difficulty in choosing only one answer in Pre-Test 1, perhaps reflecting the fact that having more than in job is common in Brazil. Other small changes were made after Pre-Test 2 and incorporated into the final version of the instrument, as presented in the Table.

## DISCUSSION

The use of standardized evaluation instruments has become a necessary practice in health sciences as a way of improving scientific knowledge. Systematized data collection reduces divergence, increases the quality of information and, as a result, allows the results to be compared. Furthermore, it produces a better basis for planning public policies and clinical conduct^31^.

Structured questionnaires are widely used instruments in epidemiological research in several areas of interest. Before considering the development of any new evaluation method, a rigorous literature review is needed to identify the existing instruments that allow the desired evaluation of the study object^33^. Regarding traumatic events experienced in childhood, a variety of instruments are available^24,36^. When identifying a suitable instrument for the objectives of the study that is not available in the language of the population to be studied, the best alternative would be to start a cross-cultural adaptation^38^.

Launching the ACE-IQ for use in Brazil, through the cross-cultural adaptation process described here, will allow the use of a WHO instrument that enables the investigation of a greater number of ACE than other instruments in Portuguese. It is aimed at setting up structured evaluations and producing comparable measures between several countries with a view to building epidemiological profiles of exposure to adversity and traumatic events in childhood.

This article presents the semantic equivalence of the ACE-IQ of the Portuguese language version currently in use in Brazil. Cross-cultural adaptation is a painstaking process, and semantic equivalence is considered only one of its stages^32^, although there is no consensus on the most appropriate way of undertaking cross-cultural adaptation, and several methodological approaches have been proposed^30–32,39,40^.

Content validity was presented as part of semantic equivalence, consisting in a judgment of the representativeness and clarity of each item of the instrument according to pre-established criteria. Concordance between the judges was evaluated regarding adequacy of the content to what the instrument is designed to measure^35,41^. Although it is an assessment pointed out as subjective by some authors^35,36^ it is a process often performed in the Health Sciences^42–44^, not exempting, however, the evaluation of other psychometric measures^45^. Good semantic equivalence was found between the Portuguese language version and the original by content validity, considering the evaluation by the expert committee and the estimation of the CVI and average intelligibility during the pre-tests.

This study should be considered in light of some limitations. The pre-tests were performed on volunteer samples not representative of the general Brazilian adult population, therefore the understanding of items or expressions may vary in samples with a distinct distribution of age, education or socioeconomic class, which can be assessed in later studies, when the questionnaire produced will be applied to other samples. The original instrument features some idiomatic expressions with no direct equivalents in Brazilian Portuguese, which required an extensive search for close alternatives between the respondents and the expert committee. Evaluating community and collective violence involves multiple aspects, usually dependent on the socio-cultural context of the investigated population and the socioeconomic/political context of the region/country, as there are items which are not relevant to the Brazilian population, such as exposure to war situations, terrorism, genocide, political and ethnic conflicts, among others. On the other hand, the investigation of community violence, extremely relevant in Brazilian studies, associated with the presence of drug trafficking and cartel and/or police actions, particularly in socially vulnerable regions directly exposed to gang fights and stray bullets, was included in the ACE-QI as collective violence. Therefore, these contexts converge in the evaluation of some sections of Brazil's population.

### Limitations of the Instrument and Recommendations

The instrument adapted in this study, like most instruments for evaluating ACE, was designed to investigate exposure to ACE in adults (aged ≥ 18 years), so memory biases may occur in data information. In addition, individuals exposed to more severe violence have increased risk of death and, therefore, the most severe spectrum of exposure cannot be captured by this form of assessment.

The ACE-IQ incorporated the investigation of other kinds of exposure, such as bullying and collective or community violence, to the study of ACE. Although more comprehensive in identifying ACE, and very useful in epidemiological research, the instrument has limitations in the way it evaluates some ACE. Therefore, it is pertinent to present some considerations and recommendations regarded as relevant both for improving the instrument and for guiding its use, analysis and interpretation of results in studies using the ACE-IQ. They are the following:

1. Age: all questions are preceded by an introductory sentence determining that only experiences which occurred” during the growth period, up to 18 years of age” are to be considered. Exposure to ACE may have a harmful differential impact depending on the age in which it happened. Young children are more physically fragile, more powerless to confront violence, more vulnerable to neglect and less capable of understanding of what is happening around them. On the other hand, adolescents tend to suffer mistreatment more intensely and severely than younger children, but they may also be able to cope with it, and/or avoid it, to some extent. Therefore, we suggest that ACE be investigated in at least two distinct age periods, childhood (up to 10 years) and adolescence (from 11 to 18 years). In addition, duration of exposure to ACE also has a direct implication on the negative consequences and should be included in the questionnaire.
2. Severity of physical abuse: in question A3, there are expressions that involve varying degrees of physical aggression (from “slapped” to “kicked” “punched” and “hit”), which generates confusion when answering since some forms of mild physical aggression can be culturally justified as disciplinary methods. An attempt to solve this difficulty was removing the expression “slapped”. Physical abuse of a child is defined as “the intentional use of physical force that results, or is highly likely to result, in damage to the child's health, survival, development, or dignity”^3,46^. Thus, we suggest adding sub-items to classify the degree of aggression (mild, moderate, intense or very intense) and to produce more robust estimates about child physical abuse.
3. Perpetration of sexual abuse: items A5-A8 present an abrupt change in the format of questions investigating the occurrence of sexual abuse, since all previous items were strictly related to the family environment. We suggest adding an introductory sentence to the session to clarify that the objective of this module is to investigate sexual abuse occurring before the age of 18 and perpetrated by any person, known or not to the victim. We also suggest adding a sub-item to all questions to identify whether or not the perpetrator is a resident of the household, as this information is relevant for understanding the impact of the various types of sexual abuse perpetrated against children and adolescents. Sexual abuse in the domestic environment tends to be repetitive, and the child is initially unaware and unable to discern the abuse. Thus, the perpetrator, who should protect and care for the child or adolescent, tends to benefit from the relationship of trust and power, taking advantage of their vulnerability, immaturity and insecurity to impose sexual assault and silence under threats and/or physical force^47^. Outside the domestic environment, sexual abuse tends to occur occasionally, with the victim being held hostage by the perpetrator only during the assault per se. In any case, sexual abuse is a traumatic phenomenon with serious consequences, and identifying the aggressors can be crucial for the implementation of preventive strategies, besides being clinically relevant for the therapeutic management of victims of sexual abuse.
4. Bullying: only the most frequent form of bullying is investigated, disregarding the multiplicity and severity of aggressions. In addition, the possibility that the victim is also an aggressor is not investigated, considering that children and adolescents who are victims of ACE can become aggressors, thus perpetuating a cycle of abuse and naturalizing violence in problem-solving. Thus, we suggest allowing multiple choice answers and investigating the perpetration of bullying.
5. Other types of ACE: understanding about ACE has changed throughout history and, as a result, new categories were defined and explored in the making of new questionnaires. Although the ACE-IQ features a wider range of AEC than the previous instruments, some adversities described in other questionnaires were not included, such as, for example, exposure to economic adversity and occurrence of physical diseases and/or serious accidents. Therefore, we suggest that a final item be included to identify the occurrence of other unevaluated traumatic events.

## CONCLUSION

As the instrument adapted in this study showed good semantic equivalence, it was launched in Brazil as a tool for epidemiological research allowing comparability between national and international studies. In addition, it can contribute to scientific knowledge about the harmful impact of ACE on the adult life of the Brazilian population, with great relevance to public health. The systematic investigation of the ACE also has clinical importance as it provides information on the patient's traumatic historical context, enabling a more comprehensive evaluation, more accurate diagnoses and more adequate treatments, thereby reducing health system costs, loss of productivity and personal suffering and disability.

Finally, it can provide relevant information to support the planning and elaboration of public policies for prevention and intervention, aiming to minimize exposure to adverse and traumatic experiences in childhood and adolescence.
