# Supplementary material for: Cross-cultural Adaptation of the Adverse Childhood Experiences International Questionnaire
Source: Rev Saude Publica. 2021 Nov 18;55:79. doi: 10.11606/s1518-8787.2021055003140 (PMC9586440; doi:10.11606/s1518-8787.2021055003140)
Supplement: Translation

# Translation: Adaptação transcultural do *Adverse Childhood Experiences International Questionnaire*

**Authors:** Flávia Garcia Pereira, Maria Carmen Viana

**Affiliations:** I Vitória ES Brasil Universidade Federal do Espírito Santo. Programa de Pós-Graduação em Saúde Coletiva. Vitória, ES, Brasil; II Vitória ES Brasil Universidade Federal do Espírito Santo. Departamento de Medicina Social. Vitória, ES, Brasil

**Keywords:** Experiências Adversas da Infância, Inquéritos e Questionários, Traduções, Adultos Sobreviventes de Eventos Adversos na Infância, Comparação Transcultural

## Abstract

**OBJETIVO:**

Traduzir, adaptar e validar o conteúdo do *Adverse Childhood Experiences International Questionnaire* para uma versão em português, vigente no Brasil, nomeada como Questionário Internacional de Experiências Adversas na Infância.

**MÉTODOS:**

Trata-se de um estudo metodológico de adaptação transcultural de instrumentos de avaliação, em que são apresentados os resultados da equivalência semântica entre o instrumento original e a versão adaptada. A equivalência semântica do instrumento envolveu as seguintes etapas: 1) duas traduções e uma síntese das traduções; 2) duas retraduções; 3) validação de conteúdo realizado por oito profissionais da área da saúde; 4) síntese das retraduções; 5) pré-testes para avaliar a aceitabilidade, a compreensão e o impacto emocional das questões; e, por fim, 6) a elaboração da versão final do instrumento.

**RESULTADOS:**

A versão adaptada demonstrou ser de fácil aplicação e compreensão e obteve boa equivalência semântica quando comparada à original. As propriedades psicométricas do instrumento devem, ainda, ser avaliadas. Limitações e recomendações para a melhoria do instrumento e para a sua utilização são apresentadas.

**CONCLUSÃO:**

O processo de adaptação transcultural do *Adverse Childhood Experiences International Questionnaire* proporcionou uma versão adaptada para o português vigente no Brasil.

## INTRODUÇÃO

Embora a história e a documentação acerca da ocorrência de maus-tratos infantis não sejam recentes^1,2^, o estudo sistemático das experiências adversas na infância (EAI) tornou-se objeto de interesse da comunidade científica a partir da década de 1990, após a publicação dos resultados do *Adverse Childhood Experiences (ACE) Study*^3,4^.

O *ACE Study*, realizado em San Diego e conduzido pelo Departamento de Medicina Preventiva da Kaiser Permanente^®^ em parceria com o Centro de Controle e Prevenção de Doenças dos Estados Unidos (CDC), é considerado um marco na investigação da exposição ao abuso e negligência durante a infância como fator de risco associado a desfechos negativos de saúde e bem-estar ao longo da vida^3,4^. A partir de sua publicação, diversos estudos epidemiológicos foram conduzidos, dando ênfase a uma crescente preocupação mundial sobre as consequências dos maus-tratos infantis, não somente imediatas, mas também a longo prazo^5–7^.

As EAI podem resultar em morbimortalidade decorrente diretamente da violência, embora, com maior frequência, as consequências envolvam danos emocionais, cognitivos e comportamentais que conferem risco aumentado, tanto para o desenvolvimento de doenças crônicas na vida adulta, como para a exposição a comportamentos de risco na adolescência^8^. Nesse sentido, diversos estudos demonstraram associações positivas e dose-dependente entre a exposição às EAI e doença coronariana, diabetes, câncer, doença pulmonar obstrutiva crônica^3^, transtornos mentais^9–11^, síndrome da fadiga crônica^12^, dor crônica^13^, tentativas de suicídio^14^, comportamento sexual de risco^15^, uso mais precoce de álcool, tabaco e outras drogas^16,17^, e mortalidade prematura^18,19^, entre outros. Além disso, desfechos negativos na vida escolar, renda e ocupação têm sido descritos^20^.

A melhor compreensão do papel deletério das EAI pode proporcionar suporte fundamental para o direcionamento e implementação de estratégias de prevenção, intervenção e tratamento adequados, tanto no estabelecimento de políticas públicas de saúde, justiça e cidadania, quanto na sensibilização, aprimoramento da formação e integração dos profissionais envolvidos na redução de maus-tratos na infância e adolescência e suas consequências^21^. Nesse sentido, o desenvolvimento de instrumentos padronizados de avaliação das EAI é fundamental para que medidas confiáveis e válidas melhorem a qualidade da informação.

Nas três últimas décadas, diversos instrumentos para a investigação das EAI foram propostos, abarcando a avaliação de diferentes categorias e contextos ambientais, com insuficiente verificação subsequente de suas propriedades psicométricas^22–24^. Vale ressaltar que, até a elaboração deste artigo, nenhum instrumento desenvolvido no Brasil foi identificado, embora alguns tenham sido adaptados para uso em populações brasileiras^25,26^ A grande variedade de instrumentos empregados tem dificultado a comparabilidade dos resultados produzidos, não somente entre contextos sociais diversos como também entre períodos diferentes na mesma população. Nessa perspectiva, a Organização Mundial da Saúde (OMS), em parceria com o CDC, elaborou o *Adverse Childhood Experiences International Questionnaire* (ACE-IQ), disponibilizando um instrumento padronizado para a avaliação e vigilância internacional das EAI e possibilitando a formulação de programas e políticas de prevenção globais de maus-tratos infantis^27,28^.

O ACE-IQ foi produzido para ser aplicado em adultos (≥ 18 anos), com o intuito de identificar e avaliar a exposição pregressa a 13 categorias diferentes de EAI, incluindo abuso sexual, emocional e físico, negligência emocional e física, violência familiar, uso de álcool/drogas, doença mental ou suicídio no âmbito domiciliar, envolvimento da família com atividades criminais, separação ou divórcio parental, violência comunitária, violência coletiva e *bullying*^27,28^. É importante destacar que todos os instrumentos de investigação de EAI coletam informações retrospectivas, sujeitas, portanto, a vieses de memória; além disso, o viés de sobrevida deve ser considerado, uma vez que violências mais graves podem levar ao óbito das vítimas.

A exposição à violência infantil é de grande relevância no campo da saúde pública, pois representa um risco ao pleno desenvolvimento infantil, com amplo impacto na vida adulta, sendo necessário estudar e comparar perfis epidemiológicos das populações afetadas. No Brasil, ainda são escassos os estudos epidemiológicos de EAI, tendo sido usados diferentes instrumentos de avaliação ou questionários não padronizados, o que dificulta a comparabilidade entre os resultados observados e o contraste com estudos internacionais. Assim, considerando-se que existe um instrumento proposto pela OMS com o intuito de estabelecer parâmetros mundiais, é pertinente a realização de uma adaptação transcultural do ACE-IQ para ser utilizada na população brasileira, já que a elaboração e desenvolvimento de novos instrumentos de avaliação é um processo complexo, que envolve diversas etapas para demonstrar propriedades psicométricas satisfatórias^29^.

A adaptação transcultural é um procedimento minucioso que analisa a utilização da linguagem, levando em consideração o contexto cultural e o estilo de vida da população para a qual o instrumento se destina. É necessário garantir as equivalências conceitual e de itens, semântica, operacional e de mensuração entre o instrumento original e o adaptado ao novo idioma^30,31^.

Com o intuito de disponibilizar um instrumento padronizado de avaliação das EAI para ser usado no Brasil, aumentando a comparabilidade com outros estudos e contribuindo para a validação do novo instrumento proposto pela OMS, foi realizada a adaptação transcultural do ACE-IQ. Neste trabalho apresenta-se a equivalência semântica da versão em português do ACE-IQ, nomeada como Questionário Internacional de Experiências Adversas na Infância (EAI-QI).

## MÉTODOS

O processo de adaptação transcultural (Figura 1) utilizado neste estudo foi baseado na metodologia adotada por Reichenheim e Moraes (2007)^32^ e nas recomendações para a adaptação do ACE-IQ disponibilizadas no site da OMS^33,34^. O ACE-IQ foi desenvolvido para ser utilizado tanto em formato autoaplicado como em entrevistas face a face^27,28^. Para o processo de adaptação transcultural, foi utilizado o questionário em forma de entrevista.

**Figura:** Fluxograma do processo de Adaptação Transcultural do Adverse Childhood Experiences International Questionnaire (ACE-IQ).

O processo de adaptação transcultural foi dividido em duas fases. A primeira fase consistiu na avaliação da equivalência conceitual e de itens, realizada pelos pesquisadores, que resultou numa revisão integrativa dos instrumentos mais utilizados nos últimos 10 anos^26^. Inicialmente, foram realizadas discussões temáticas informais junto ao Centro de Estudos e Pesquisa em Epidemiologia Psiquiátrica da Universidade Federal do Espírito Santo (Cepep/Ufes), coordenado por MCV, e formado por mestres e doutores na área de saúde coletiva, a fim de discutir e verificar a relevância dos itens do instrumento original para captar as informações sobre as EAI no contexto brasileiro. As discussões foram baseadas nos principais questionários e inquéritos conduzidos no contexto internacional e local, identificados por meio de um estudo de revisão sistemática das publicações disponíveis até 2003^24^.

Em seguida, deu-se início à segunda fase da adaptação transcultural que consistiu na avaliação da equivalência semântica. Essa fase foi subdividida em cinco etapas. Inicialmente (Etapa 1), foram obtidas duas traduções (T1 e T2) do instrumento original em inglês para o português, realizadas de forma independente por tradutores cuja língua materna é o português. Um dos tradutores tem formação profissional em medicina e experiência no campo da violência contra a mulher e o outro tem formação em nutrição e experiência profissional no campo de doenças crônicas não transmissíveis. Ainda na Etapa 1, as discrepâncias nas traduções foram identificadas pelos pesquisadores, solicitando-se aos tradutores que chegassem a um consenso, para, em seguida, obter-se uma versão-síntese (S1) do questionário em português (Etapa 1).

Na segunda etapa (Etapa 2), S1 foi submetida a duas retraduções (para o inglês) por tradutores independentes cuja língua materna é o inglês, sendo uma delas realizada por uma empresa especializada (R1) e a outra por uma professora da língua inglesa (R2).

Para a realização da terceira etapa (Etapa 3), foram convidados 13 profissionais graduados em diferentes áreas, para compor o comitê de especialistas. O convite foi enviado através de uma carta explicativa, via e-mail, sobre as funções do comitê, contendo, ainda, informações sucintas acerca do questionário ACE-IQ e dos métodos empregados na adaptação transcultural de instrumentos. Oito profissionais aceitaram participar da Etapa 3, sendo seis graduados em medicina, um em psicologia e um em artes, com especialização em antropologia. Os outros sete têm doutorado em diferentes áreas do conhecimento (epidemiologia/saúde coletiva, fisiologia, psiquiatria, psicofarmacologia). A composição de um comitê de especialistas em diferentes áreas do conhecimento tem por objetivo atestar a compreensão ampla do questionário, tendo em vista sua aplicabilidade na avaliação da população geral^35^.

Ao comitê de especialistas foi delegada a avaliação das equivalências semânticas, conceitual e idiomática entre o instrumento original e as duas retraduções obtidas, a fim de verificar a correspondência literal de cada item traduzido e avaliar a correspondência com o significado original, levando em consideração o sentido que algumas palavras assumem no novo contexto cultural no qual o instrumento será empregado. Esse processo tem como propósito identificar possíveis erros ocorridos nas etapas anteriores e obter sugestões para melhor adaptação de uma primeira versão do questionário (Q1) em português. Todos os membros do comitê tiveram acesso às versões produzidas em cada etapa (S1, R1 e R2), além do questionário original em inglês, para comparação.

Foi solicitado aos especialistas que avaliassem, para cada item e para o questionário como um todo, a adequação dos seguintes quesitos: correspondência da S1 com a versão original, pertinência/importância, clareza, vocabulário, objetividade, aplicabilidade ao contexto brasileiro e sequência instrucional dos itens. Para a avaliação desses quesitos, adotou-se uma escala Likert de 1 a 4 (1 = adequado; 2 = adequado com poucas alterações; 3=adequado com muitas alterações e 4 = inadequado). Mediante análise da avaliação dos itens pelo comitê de especialistas, foi calculado, de forma adaptada, o índice de validade de conteúdo (IVC) [onde IVC = (número de respostas 1 e 2)/(número total de especialistas)], sendo considerado índice adequado o valor acima de 0,8^35^. Também foi solicitado aos especialistas que avaliassem a correspondência das retraduções (R1 e R2) de cada item em relação ao questionário original (1 = inalterado; 2 = pouco alterado; 3 = muito alterado e 4 = completamente alterado)^35,36^.

A quarta etapa (Etapa 4) consistiu na elaboração de uma versão síntese das retraduções (RS1) e na versão Q1. A versão síntese foi produzida a partir da comparação das duas retraduções avaliadas pelo comitê de especialistas, por um terceiro tradutor (R3), proficiente e fluente nos dois idiomas, com formação em medicina e amplo conhecimento na temática sobre experiências adversas na infância, além de atuante na área da saúde coletiva (MCV). A versão Q1 foi, então, produzida considerando o julgamento da adequação de cada item do questionário pelo comitê de especialistas, e suas propostas de melhor adequação em itens considerados inadequados, desde que fossem pertinentes ao contexto original do ACE-IQ pelos pesquisadores, de acordo com os documentos de instrução da OMS^34^.

A última etapa (Etapa 5) da equivalência semântica constituiu-se na realização de um pré-teste (Pré-teste 1) com a versão Q1 em uma amostra de voluntários estudantes e funcionários de um programa de pós-graduação, composta por nove mulheres e dois homens, com o intuito de avaliar a aceitabilidade e a compreensão dos itens e a reação emocional dos entrevistados aos temas abordados^32^. A idade dos participantes variou de 28 a 48 anos, com escolaridade do ensino médio ao doutorado. O questionário (Q1) foi aplicado de forma padronizada, através de entrevistas face a face conduzidas por um dos pesquisadores (FGP).

Uma escala verbal numérica adaptada^25^ foi utilizada pelos entrevistados para que avaliassem a facilidade de compreensão de cada item do questionário. A pergunta norteadora para todos os itens era “Você entendeu o que foi perguntado?”, sendo três as respostas possíveis: 1 = não entendi nada; 2 = entendi, mas tenho dúvidas; 3 = entendi perfeitamente e não tenho dúvidas. Também foi solicitado aos entrevistados que explicassem o que entenderam sobre cada item do questionário, a fim de avaliar se cada questão reproduzia o sentido do item original. Ao final da aplicação do questionário, os participantes responderam às seguintes perguntas, ainda em entrevista face a face com a pesquisadora (FGP): a) todos os itens do instrumento apresentam opções de resposta satisfatórias?; b) os termos utilizados lhe são familiares?; c) você considera que algum termo ou trecho é ambíguo ou difícil de entender?; e d) sobre os itens que não foram compreendidos, ou de uma maneira geral, você gostaria de apresentar alguma sugestão para melhorar o questionário? As perguntas eram abertas e todas as respostas foram exploradas e anotadas.

A partir das considerações e dúvidas obtidas nessa etapa, uma segunda versão (Q2) foi produzida e apresentada a dez novos voluntários, sendo oito mulheres e dois homens. Esse grupo foi composto por estudantes e funcionários de um programa de pós-graduação em saúde coletiva com idade entre 19 e 42 anos, escolaridade variando do nível médio (técnico profissionalizante) até o doutorado. Foram realizadas entrevistas face a face, em que os participantes fizeram a leitura do questionário em voz alta explicando o que entenderam a respeito de cada item (Pré-teste 2). A versão Q2 foi, então, ajustada e produzida a versão final do ACE-IQ adaptado para uso no Brasil, denominado “Questionário Internacional de Experiências Adversas na Infância (EAI-QI)”, disponível no Quadro. Foram mantidas a numeração e a codificação de todos os itens conforme o instrumento original, para facilitar a comparação com outros estudos e a compilação de múltiplos bancos de dados (Quadro).

**Quadro:** Versão original do Adverse Childhood Experiences International Questionnaire (ACE-IQ) e versões produzidas em português e adaptadas para uso no Brasil.

	VERSÃO ORIGINAL	PRIMEIRA VERSÃO (Q1) Aplicada no Pré-teste 1	VERSÃO FINAL DO QUESTIONÁRIO Considerando os resultados do Pré-teste 2
	Adverse Childhood Experiences International Questionnaire (ACE-IQ)	Questionário Internacional de Experiências Adversas na Infância (EAI-QI)	Questionário Internacional de Experiências Adversas na Infância (EAI-QI)
0	***DEMOGRAPHIC INFORMATION***	**INFORMAÇÕES DEMOGRÁFICAS**	**INFORMAÇÕES DEMOGRÁFICAS**
0.1 [C1]	*Sex (Record Male/Female as observed)* *[ ] Male* *[ ] Female*	Sexo (Marque de acordo com o observado) [ ] Masculino [ ] Feminino	Sexo (Marque de acordo com o observado) [ ] Masculino [ ] Feminino
0.2 [C2]	*What is your date of birth?* *Day [ ][ ] Month [ ][ ] Year [ ][ ][ ][ ]* *Unknown (Go to Q.C3)*	Qual a data do seu nascimento? Dia [ ] mês [ ] ano [ ][ ][ ][ ] Não sabe/não informou [ ] ***(Vá para Q.C3)***	Qual a data do seu nascimento? Dia [ ] mês [ ] ano [ ][ ][ ][ ] Não sabe (não sei)/não informou (não quero informar) [ ] ***(Vá para Q.C3)***
0.3 [C3]	*How old are you?* *[ ] [ ]*	Qual é a sua idade? [ ] [ ]	Qual é a sua idade? [ ] [ ]
0.4 [C.4]	*What is your [insert relevant ethnic group/racial group/cultural group/others] background?* *[Locally defined]* *[Locally defined]* *[Locally defined]* *Refused*	Qual das seguintes alternativas você considera como a sua raça/cor de pele? [ ] Branca [ ] Preta [ ] Parda [ ] Amarela [ ] Indígena [ ] Outras: _____________________________ [ ] Não quis responder	Qual das seguintes alternativas você considera como a sua raça/cor da pele? [ ] Branca [ ] Preta [ ] Parda [ ] Amarela [ ] Indígena [ ] Outras: _____________________________ [ ] Não declarada/Não quero declarar [ ] Não sabe informar/Não sei informar
0.5 [C5]	*What is the highest level of education you have completed?* *No formal schooling* *Less than primary school* *Primary school completed* *Secondary/High school completed* *College/University completed* *Post graduate degree* *Refused*	Qual é o seu mais alto nível de escolaridade? [ ] Não frequentou a escola [ ] Ensino Fundamental I (até 5° ano ou 4ᵃ série) [ ] Ensino Fundamental II incompleto (até 9° ano ou 8ᵃ série) [ ] Ensino Fundamental completo [ ] Ensino Médio incompleto [ ] Ensino Médio completo [ ] Ensino Superior incompleto [ ] Ensino Superior completo [ ] Pós-Graduação [ ] Não quis responder	Qual é o seu nível de escolaridade mais alto? [ ] Não frequentou a escola [ ] Ensino Fundamental I (até 5° ano ou 4ᵃ série) [ ] Ensino Fundamental II incompleto (até 9° ano ou 8ᵃ série) [ ] Ensino Fundamental completo [ ] Ensino Médio incompleto [ ] Ensino Médio completo [ ] Ensino Superior incompleto [ ] Ensino Superior completo [ ] Pós-Graduação [ ] Não quis responder/Não quero responder
0.6 [C6]	*Which of the following best describes your main work status over the last 12 months?* *Government employee* *Non-government employee* *Self-employed* *Non-paid* *Student* *Homemaker* *Retired* *Unemployed (able to work)* *Unemployed (unable to work)* *Refused*	Quais das seguintes opções melhor descrevem a sua situação de trabalho nos últimos 12 meses? (assinale todas as alternativas que se aplicam) [ ] Funcionário público [ ] Empregado do setor privado [ ] Trabalhador autônomo/trabalha por conta própria/Empregador [ ] Trabalho não remunerado/trabalho voluntário [ ] Estudante [ ] Dona de casa/Trabalho doméstico não remunerado [ ] Aposentado(a) [ ] Desempregado(a) – com capacidade de trabalhar [ ] Desempregado(a) – sem capacidade de trabalhar [ ] Outros:_____________________________ [ ] Não quis responder	Quais das seguintes opções melhor descrevem a sua principal situação de trabalho nos últimos 12 meses? (assinalar todas as alternativas que se aplicam) [ ] Funcionário público/Vínculo com o setor público [ ] Empregado do setor privado [ ] Trabalhador autônomo/trabalha por conta própria/Empregador [ ] Trabalho não remunerado/trabalho voluntário [ ] Estudante [ ] Dona de casa/Trabalho doméstico não remunerado [ ] Aposentado(a) [ ] Desempregado(a) – com capacidade de trabalhar [ ] Desempregado(a) – sem capacidade de trabalhar [ ] Outros:_____________________________ [ ] Não quis responder/Não quero responder
0.7 [C7]	*What is your civic status?* *Married (Go to Q.M2)* *Living as couple* *Divorced or separated* *Single* *Widowed (Go to Q.M2)* *Other* *Refused*	Qual o seu estado civil? [ ] Casado(a) ***(Vá para Q.M2)*** [ ] Vive com companheiro(a) [ ] Divorciado(a)/separado(a) [ ] Solteiro(a) [ ] Viúvo(a) ***(Vá para Q.M2)*** [ ] Outro: ______________________________ [ ] Não quis responder	Qual o seu estado civil? [ ] Casado(a) ***(Vá para Q.M2)*** [ ] Vive com companheiro(a) [ ] Divorciado(a)/separado(a) [ ] Solteiro(a) [ ] Viúvo(a) ***(Vá para Q.M2)*** [ ] Outro: ______________________________ [ ] Não quis responder/Não quero responder
**1**	***MARRIAGE***	**CASAMENTO**	**CASAMENTO**
1.1 [M1]	*Have you ever been married?* *Yes* *No (Go to Q.M5)* *Refused*	Você já foi casado(a)? [ ] Sim [ ] Não ***(Vá para Q.M5)*** [ ] Não quis responder	Você já foi casado(a)? [ ] Sim [ ] Não ***(Vá para Q.M5)*** [ ] Não quis responder/Não quero responder
1.2 [M2]	*At what age were you first married?* *Age [ ][ ]* *Refused*	Com que idade você se casou pela primeira vez? Idade [ ] [ ] [ ] Não quis responder	Com que idade você se casou pela primeira vez? Idade [ ] [ ] [ ] Não quis responder/Não quero responder
1.3 [M3]	*At the time of your first marriage did you yourself choose your husband/wife?* *Yes (Go to Q.M5)* *No* *Don't know/Not sure* *Refused*	Quando você se casou pela primeira vez, foi você mesmo(a) quem escolheu seu marido/esposa? [ ] Sim ***(Vá para Q.M5)*** [ ] Não [ ] Não sabe/Não tem certeza [ ] Não quis responder	Quando você se casou pela primeira vez, foi você mesmo(a) quem escolheu seu marido/esposa? [ ] Sim ***(Vá para Q.M5)*** [ ] Não [ ] Não sabe (Não sei)/Não tem certeza (Não tenho certeza) [ ] Não quis responder/Não quero responder
1.4 [M4]	*At the time of your first marriage if you did not choose your husband/wife yourself, did you give your consent to the choice?* *Yes* *No* *Refused*	Quando você se casou pela primeira vez, se não foi você quem escolheu seu marido/esposa, você concordou com a escolha? [ ] Sim [ ] Não [ ] Não quis responder	Quando você se casou pela primeira vez, se não foi você quem escolheu seu marido/esposa, você concordou com a escolha? [ ] Sim [ ] Não [ ] Não quis responder/Não quero responder
1.5 [M5]	*If you are a mother or father what was your age when your first child was born?* *Age [ ][ ]* *Not applicable* *Refused*	Se você já teve filhos, qual era sua idade quando nasceu seu(sua) primeiro(a) filho(a)? Idade [ ] [ ] Não se aplica [ ] [ ] Não quis responder	Se você já teve filhos, qual era sua idade quando nasceu seu(sua) primeiro(a) filho(a)? Idade [ ] [ ] Não se aplica [ ] [ ] Não quis responder/Não quero responder
**2**	***RELATIONSHIP WITH PARENTS/GUARDIANS*** *When you were growing up, during the first 18 years of your life…*	**RELACIONAMENTO COM OS PAIS OU RESPONSÁVEIS** Durante o período de crescimento, nos primeiros 18 anos de sua vida…	**RELACIONAMENTO COM OS PAIS OU RESPONSÁVEIS** A partir de agora, todas as perguntas estão relacionadas ao período em que você estava crescendo, até os seus 18 anos.
2.1 [P1]	*Did your parents/guardians understand your problems and worries?* *Always* *Most of the time* *Sometimes* *Rarely* *Never* *Refused*	Seus pais/responsáveis compreendiam seus problemas e preocupações? [ ] Sempre [ ] Na maioria das vezes [ ] Às vezes [ ] Raramente [ ] Nunca [ ] Não quis responder	Seus pais/responsáveis compreendiam seus problemas e preocupações? [ ] Sempre [ ] Na maioria das vezes [ ] Às vezes [ ] Raramente [ ] Nunca [ ] Não quis responder/Não quero responder
2.2 [P2]	*Did your parents/guardians* ***really*** *know what you were doing with your free time when you were not at school or work?* *Always* *Most of the time* *Sometimes* *Rarely* *Never* *Refused*	Seus pais/responsáveis **realmente** sabiam o que você estava fazendo no seu tempo livre, quando não estava na escola ou no trabalho? [ ] Sempre [ ] Na maioria das vezes [ ] Às vezes [ ] Raramente [ ] Nunca [ ] Não quis responder	Seus pais/responsáveis **realmente** sabiam o que você estava fazendo no seu tempo livre, quando não estava na escola ou no trabalho? [ ] Sempre [ ] Na maioria das vezes [ ] Às vezes [ ] Raramente [ ] Nunca [ ] Não quis responder/Não quero responder
3			
3.1 [P3]	*How often did your parents/guardians **not** give you enough food even when they could easily have done so?* *Many times* *A few times* *Once* *Never* *Refused*	Com que frequência seus pais/responsáveis **não** lhe davam comida suficiente, mesmo que pudessem facilmente fazer isso? [ ] Muitas vezes [ ] Poucas vezes [ ] Uma vez [ ] Nunca [ ] Não quis responder	Com que frequência seus pais/responsáveis **não** lhe davam comida suficiente, mesmo que pudessem facilmente oferecer um alimento para você? [ ] Muitas vezes [ ] Poucas vezes [ ] Uma vez [ ] Nunca [ ] Não quis responder/Não quero responder
3.2 [P4]	*Were your parents/guardians too drunk or intoxicated by drugs to take care of you?* *Many times* *A few times* *Once* *Never* *Refused*	Seus pais/responsáveis ficavam muito embriagados ou sob o efeito de drogas quando cuidavam de você? [ ] Muitas vezes [ ] Poucas vezes [ ] Uma vez [ ] Nunca [ ] Não quis responder	Seus pais/responsáveis ficavam muito embriagados ou sob o efeito de drogas quando cuidavam de você? [ ] Muitas vezes [ ] Poucas vezes [ ] Uma vez [ ] Nunca [ ] Não quis responder/Não quero responder
3.3 [P5]	*How often did your parents/guardians* ***not*** *send you to school even when it was available?* *Many times* *A few times* *Once* *Never* *Refused*	Com que frequência seus pais/responsáveis **não** lhe mandavam para a escola, mesmo que devessem fazer isso? [ ] Muitas vezes [ ] Poucas vezes [ ] Uma vez [ ] Nunca [ ] Não quis responder	Com que frequência seus pais/responsáveis **não** lhe mandavam para a escola, mesmo que tivessem a obrigação de fazer isso? [ ] Muitas vezes [ ] Poucas vezes [ ] Uma vez [ ] Nunca [ ] Não quis responder/Não quero responder
4	***FAMILY ENVIRONMENT*** *When you were growing up, during the first 18 years of your life…*	**AMBIENTE FAMILIAR** Durante o período de crescimento, nos primeiros 18 anos de sua vida…	**AMBIENTE FAMILIAR** Nos primeiros 18 anos da sua vida…
4.1 [F1]	*Did you live with a household member who was a problem drinker or alcoholic, or misused street or prescription drugs?* *Yes* *No* *Refused*	Você morou com alguém que tinha problemas com álcool ou era alcoólatra, ou que abusava de drogas ilícitas ou de medicamentos controlados? [ ] Sim [ ] Não [ ] Não quis responder	Você morou com alguém que tinha problemas com álcool ou era alcoólatra, ou que abusava de drogas ilícitas ou de medicamentos controlados? [ ] Sim [ ] Não [ ] Não quis responder/Não quero responder
4.2 [F2]	*Did you live with a household member who was depressed, mentally ill or suicidal?* *Yes* *No* *Refused*	Você morou com alguém que estava deprimido, ou tinha alguma doença mental ou intenção suicida? [ ] Sim [ ] Não [ ] Não quis responder	Você morou com alguém que estava deprimido, ou tinha alguma doença mental ou intenção suicida? [ ] Sim [ ] Não [ ] Não quis responder/Não quero responder
4.3 [F3]	*Did you live with a household member who was ever sent to jail or prison?* *Yes* *No* *Refused*	Você morou com alguém que alguma vez tenha sido levado pra cadeia ou mandado pra prisão? [ ] Sim [ ] Não [ ] Não quis responder	Você morou com alguém que alguma vez tenha sido levado pra cadeia ou mandado pra prisão? [ ] Sim [ ] Não [ ] Não quis responder/Não quero responder
4.4 [F4]	*Were your parents ever separated or divorced?* *Yes* *No* *Not applicable* *Refused*	Nesse período, seus pais alguma vez se separaram ou se divorciaram? [ ] Sim [ ] Não [ ] Não se aplica [ ] Não quis responder	Nesse período (até os seus 18 anos) seus pais alguma vez se separaram ou se divorciaram? [ ] Sim [ ] Não [ ] Não se aplica [ ] Não quis responder/Não quero responder
4.5 [F5]	*Did your mother, father or guardian die?* *Yes* *No* *Don't know/Not sure* *Refused*	Sua mãe, pai ou responsável faleceram (nesse período)? [ ] Sim [ ] Não [ ] Não sabe/Não tem certeza [ ] Não quis responder	Sua mãe, pai ou responsável faleceram (nesse período)? [ ] Sim [ ] Não [ ] Não sabe/Não tem certeza [ ] Não quis responder/Não quero responder
	*These next questions are about certain things you may actually have heard or seen **IN YOUR HOME**. These are things that may have been done to another household member but not necessarily to you.* *When you were growing up, during the first 18 years of your life…*	As próximas perguntas são sobre algumas situações/coisas que você pode ter ouvido ou visto **EM SUA CASA**. São situações/coisas que podem ter acontecido com outros moradores da sua casa, não necessariamente com você. Durante o período de crescimento, nos primeiros 18 anos de sua vida…	As próximas perguntas estão relacionadas a algumas situações que você pode ter ouvido ou visto **EM SUA CASA**. São situações que podem ter acontecido **COM OS MORADORES DA SUA CASA, NÃO COM VOCÊ**. Nos primeiros 18 anos da sua vida…
4.6 [F6]	*Did you see or hear a parent or household member in your home being yelled at, screamed at, sworn at, insulted or humiliated?* *Many times* *A few times* *Once* *Never* *Refused*	Você viu ou ouviu algum de seus pais ou alguém que morava na sua casa recebendo gritos ou berros, ou sendo xingado, insultado ou humilhado? [ ] Muitas vezes [ ] Poucas vezes [ ] Uma vez [ ] Nunca [ ] Não quis responder	Você viu ou ouviu alguém que morava na sua casa recebendo gritos ou berros, ou sendo xingado, insultado ou humilhado? [ ] Muitas vezes [ ] Poucas vezes [ ] Uma vez [ ] Nunca [ ] Não quis responder/Não quero responder
4.7 [F7]	*Did you see or hear a parent or household member in your home being slapped, kicked, punched or beaten up?* *Many times* *A few times* *Once* *Never* *Refused*	Você viu ou ouviu algum de seus pais ou alguém que morava na sua casa sendo estapeado, chutado, socado ou surrado? [ ] Muitas vezes [ ] Poucas vezes [ ] Uma vez [ ] Nunca [ ] Não quis responder	Você viu ou ouviu alguém que morava na sua casa sendo estapeado, chutado, socado ou surrado? [ ] Muitas vezes [ ] Poucas vezes [ ] Uma vez [ ] Nunca [ ] Não quis responder/Não quero responder
4.8 [F8]	*Did you see or hear a parent or household member in your home being hit or cut with an object, such as a stick (or cane), bottle, club, knife, whip etc.?* *Many times* *A few times* *Once* *Never* *Refused*	Você viu ou ouviu algum de seus pais ou alguém que morava na sua casa ser atingido ou cortado com algum objeto, como uma vara (ou bengala), garrafa, porrete, faca, chicote, etc? [ ] Muitas vezes [ ] Poucas vezes [ ] Uma vez [ ] Nunca [ ] Não quis responder	Você viu ou ouviu alguém que morava na sua casa ser agredido ou cortado com algum objeto, como uma vara (ou bengala), garrafa, porrete, faca, chicote, ou algum outro objeto? [ ] Muitas vezes [ ] Poucas vezes [ ] Uma vez [ ] Nunca [ ] Não quis responder/Não quero responder
5	*These next questions are about certain things* ***YOU*** *may have experienced.* *When you were growing up, during the first 18 years of your life…*	Estas próximas perguntas são sobre certas coisas que **VOCÊ** pode ter vivenciado. Durante o período de crescimento, nos primeiros 18 anos de sua vida…	As próximas perguntas estão relacionadas a algumas situações que **VOCÊ** pode ter vivenciado. Nos primeiros 18 anos da sua vida…
5.1 [A1]	*Did a parent, guardian or other household member yell, scream or swear at you, insult or humiliate you?* *Many times* *A few times* *Once* *Never* *Refused*	Algum de seus pais ou responsáveis ou alguém que morava na sua casa gritou, berrou, xingou, insultou ou humilhou você? [ ] Muitas vezes [ ] Poucas vezes [ ] Uma vez [ ] Nunca [ ] Não quis responder	Algum de seus pais ou responsáveis ou alguém que morava na sua casa gritou, berrou, xingou, insultou ou humilhou você? [ ] Muitas vezes [ ] Poucas vezes [ ] Uma vez [ ] Nunca [ ] Não quis responder/Não quero responder
5.2 [A2]	*Did a parent, guardian or other household member threaten to, or actually, abandon you or throw you out of the house?* *Many times* *A few times* *Once* *Never* *Refused*	Algum de seus pais ou responsáveis ou alguém que morava na sua casa ameaçou abandonar ou expulsar você de casa, ou de fato fez isso? [ ] Muitas vezes [ ] Poucas vezes [ ] Uma vez [ ] Nunca [ ] Não quis responder	Algum de seus pais ou responsáveis ou alguém que morava na sua casa **ameaçou** abandonar ou expulsar você de casa, **ou de fato fez isso**? [ ] Muitas vezes [ ] Poucas vezes [ ] Uma vez [ ] Nunca [ ] Não quis responder/Não quero responder
5.3 [A3]	*Did a parent, guardian or other household member spank, slap, kick, punch or beat you up?* *Many times* *A few times* *Once* *Never* *Refused*	Algum de seus pais ou responsáveis ou alguém que morava na sua casa bateu, deu tapas, chutou, socou ou surrou você? [ ] Muitas vezes [ ] Poucas vezes [ ] Uma vez [ ] Nunca [ ] Não quis responder	Algum de seus pais ou responsáveis ou alguém que morava na sua casa bateu, chutou, socou ou surrou você? [ ] Muitas vezes [ ] Poucas vezes [ ] Uma vez [ ] Nunca [ ] Não quis responder/Não quero responder
			Qual a intensidade dessas agressões? [ ] Leve [ ] Moderada [ ] Intensa [ ] Muito intensa [ ] Não quis responder/Não quero responder
5.4 [A4]	*Did a parent, guardian or other household member hit or cut you with an object, such as a stick (or cane), bottle, club, knife, whip etc?* *Many times* *A few times* *Once* *Never* *Refused*	Algum de seus pais ou responsáveis ou alguém que morava na sua casa atingiu ou cortou com algum objeto, como uma vara (ou bengala), garrafa, porrete, faca, chicote, etc? [ ] Muitas vezes [ ] Poucas vezes [ ] Uma vez [ ] Nunca [ ] Não quis responder	Algum de seus pais ou responsáveis ou alguém que morava na sua casa agrediu ou cortou você com algum objeto, como uma vara (ou bengala), garrafa, porrete, faca, chicote, ou algum outro objeto? [ ] Muitas vezes [ ] Poucas vezes [ ] Uma vez [ ] Nunca [ ] Não quis responder/Não quero responder
			As próximas perguntas são sobre situações de assédio/molestação sexual que VOCÊ possa ter vivenciado até os 18 anos…
5.5 [A5]	*Did someone touch or fondle you in a sexual way when you did not want them to?* *Many times* *A few times* *Once* *Never* *Refused*	Alguém tocou ou acariciou você de uma forma sexual sem que você quisesse? [ ] Muitas vezes [ ] Poucas vezes [ ] Uma vez [ ] Nunca [ ] Não quis responder	Algum de seus pais ou responsáveis ou alguém que morava na sua casa ou algum conhecido ou desconhecido **tocou ou acariciou** você de uma forma sexual sem que você quisesse? [ ] Muitas vezes [ ] Poucas vezes [ ] Uma vez [ ] Nunca [ ] Não quis responder/Não quero responder
			Foi alguém que morava na sua casa? [ ] Sim [ ] Não [ ] Não quis responder/Não quero responder
5.6 [A6]	*Did someone make you touch their body in a sexual way when you did not want them to?* *Many times* *A few times* *Once* *Never* *Refused*	Alguém fez com que você tocasse o corpo dele(a) de uma forma sexual sem que você quisesse fazer isso? [ ] Muitas vezes [ ] Poucas vezes [ ] Uma vez [ ] Nunca [ ] Não quis responder	Algum de seus pais ou responsáveis ou alguém que morava na sua casa ou algum conhecido ou desconhecido **fez com que você tocasse** o corpo dele(a) de uma forma sexual sem que você quisesse fazer isso? [ ] Muitas vezes [ ] Poucas vezes [ ] Uma vez [ ] Nunca [ ] Não quis responder/Não quero responder
			Foi alguém que morava na sua casa? [ ] Sim [ ] Não [ ] Não quis responder/Não quero responder
5.7 [A7]	*Did someone attempt oral, anal, or vaginal intercourse with you when you did not want them to?* *Many times* *A few times* *Once* *Never* *Refused*	Alguém tentou fazer sexo oral, anal ou vaginal com você sem que você quisesse? [ ] Muitas vezes [ ] Poucas vezes [ ] Uma vez [ ] Nunca [ ] Não quis responder	Algum de seus pais ou responsáveis ou alguém que morava na sua casa ou algum conhecido ou desconhecido **tentou fazer** sexo oral, anal ou vaginal com você sem que você quisesse? [ ] Muitas vezes [ ] Poucas vezes [ ] Uma vez [ ] Nunca [ ] Não quis responder/Não quero responder
			Foi alguém que morava na sua casa? [ ] Sim [ ] Não [ ] Não quis responder/Não quero responder
5.8 [A8]	*Did someone actually have oral, anal, or vaginal intercourse with you when you did not want them to?* *Many times* *A few times* *Once* *Never* *Refused*	Alguém já fez sexo oral, anal ou vaginal com você sem que você quisesse? [ ] Muitas vezes [ ] Poucas vezes [ ] Uma vez [ ] Nunca [ ] Não quis responder	Algum de seus pais ou responsáveis ou alguém que morava na sua casa ou algum conhecido ou desconhecido **já fez** sexo oral, anal ou vaginal com você sem que você quisesse? [ ] Muitas vezes [ ] Poucas vezes [ ] Uma vez [ ] Nunca [ ] Não quis responder/Não quero responder
			Foi alguém que morava na sua casa? [ ] Sim [ ] Não [ ] Não quis responder/Não quero responder
**6**	***PEER VIOLENCE***	**VIOLÊNCIA ENTRE PARES**	
	*These next questions are about BEING BULLIED when you were growing up.* *Bullying is when a young person or group of young people say or do bad and unpleasant things to another young person. It is also bullying when a young person is teased a lot in an unpleasant way or when a young pers on is left out of things on purpose. It is not bullying when two young people of about the same strength or power argue or fight or when teasing is done in a friendly and fun way. When you were growing up, during the first 18 years of your life…*	As próximas perguntas são sobre **SOFRER** ***BULLYING*** quando você estava crescendo. O *bullying* ocorre quando um jovem (criança ou adolescente) ou um grupo de jovens diz ou faz coisas ruins ou desagradáveis para outro jovem (criança ou adolescente). Também é *bullying* quando uma pessoa jovem é provocada de forma desagradável ou quando é deixada de fora das atividades de propósito. Não é *bullying* quando dois jovens com a mesma força ou poder discutem ou brigam ou quando a provocação ocorre de forma amistosa e divertida. Durante o período de crescimento, nos primeiros 18 anos de sua vida…	As próximas perguntas estão relacionadas ao ***BULLYING***. O *bullying* ocorre quando um jovem ou um grupo de jovens diz ou faz coisas ruins ou desagradáveis para outro jovem. Também é *bullying* quando uma pessoa jovem é provocada de forma desagradável ou quando é deixada de fora das atividades de propósito. Não é *bullying* quando dois jovens com a mesma força ou poder discutem ou brigam ou quando a provocação ocorre de forma amistosa e divertida. Nos primeiros 18 anos da sua vida…
6.1 [V1]	*How often were you bullied?* *Many times* *A few times* *Once* *Never (Go to Q.V3)* *Refused*	Com que frequência você sofria *bullying*? [ ] Muitas vezes [ ] Poucas vezes [ ] Uma vez [ ] Nunca *(****Vá para Q.V3)*** [ ] Não quis responder	Com que frequência você sofria *bullying*? [ ] Muitas vezes [ ] Poucas vezes [ ] Uma vez [ ] Nunca *(****Vá para Q.V3)*** [ ] Não quis responder/Não quero responder
6.2 [V2]	*How were you bullied most often?* *I was hit, kicked, pushed, shoved around, or locked indoors* *I was made fun of because of my race, nationality or colour* *I was made fun of because of my religion* *I was made fun of with sexual jokes, comments, or gestures* *I was left out of activities on purpose or completely ignored* *I was made fun of because of how my body or face looked* *I was bullied in some other way* *Refused*	Qual era a forma mais frequente de *bullying* que você sofria? [ ] Me batiam, esbarravam em mim, era chutado(a), empurrado(a), ou trancado(a) em lugares fechados [ ] Era zoado(a) (Riam de mim/me zoavam) por causa da minha raça, nacionalidade ou cor de pele [ ] Era zoado(a) (Riam de mim/me zoavam) por causa da minha religião [ ] Era zoado(a) (Riam de mim/me zoavam) por meio de brincadeiras ou comentários de cunho sexual, ou gestos obcenos [ ] Era excluído(a) de atividades de propósito ou completamente ignorado(a) [ ] Era zoado(a) (Riam de mim/me zoavam) por causa da aparência do meu corpo ou do meu rosto [ ] Eu sofria alguma outra forma de *bullying* [ ] Não quis responder	Qual era a forma **mais frequente** de *bullying* que você sofria? **(Assinalar apenas uma opção)** [ ] Me batiam, esbarravam em mim, era chutado(a), empurrado(a), ou trancado(a) em lugares fechados [ ] Era zoado(a) (debochavam de mim) por causa da minha raça, nacionalidade ou cor da pele [ ] Era zoado(a) (debochavam de mim) por causa da minha religião [ ] Era zoado(a) (debochavam de mim) por meio de brincadeiras ou comentários de cunho sexual, ou gestos obcenos [ ] Era excluído(a) de atividades de propósito ou completamente ignorado(a) [ ] Era zoado(a) (debochavam de mim) por causa da aparência do meu corpo ou do meu rosto [ ] Eu sofria alguma outra forma de *bullying* [ ] Não quis responder/Não quero responder
	*This next question is about **PHYSICAL FIGHTS**. A physical fight occurs when two young people of about the same strength or power choose to fight each other.* *When you were growing up, during the first 18 years of your life…*	A próxima pergunta é sobre **BRIGAS FÍSICAS**. Uma briga física ocorre quando dois jovens com aproximadamente a mesma força ou poder escolhem brigar um contra o outro. Durante o período de crescimento, nos primeiros 18 anos de sua vida…	A próxima pergunta é sobre **BRIGAS FÍSICAS**. Uma briga física ocorre quando dois jovens com aproximadamente a mesma força ou poder escolhem brigar um contra o outro. Durante o período de crescimento, nos primeiros 18 anos da sua vida…
6.3 [V3]	*Did you see or hear someone being beaten up in real life?* *Many times* *A few times* *Once* *Never* *Refused*	Com que frequência você se envolvia em uma briga física? [ ] Muitas vezes [ ] Poucas vezes [ ] Uma vez [ ] Nunca [ ] Não quis responder	Com que frequência você se envolvia em uma briga física? [ ] Muitas vezes [ ] Poucas vezes [ ] Uma vez [ ] Nunca [ ] Não quis responder/Não quero responder
7	***WITNESSING COMMUNITY VIOLENCE*** *These next questions are about how often, when you were a child,* ***YOU*** *may have seen or heard certain things in your* ***NEIGHBOURHOOD OR COMMUNITY*** *(not in your home or on TV, movies, or the radio).* *When you were growing up, during the first 18 years of your life…*	**VIOLÊNCIA NA COMUNIDADE** As próximas perguntas são sobre a frequência com que **VOCÊ** viu ou ouviu certas coisas na sua **VIZINHANÇA OU COMUNIDADE** quando você era criança (não na sua casa ou na TV, rádio ou em filmes). Durante o período de crescimento, nos primeiros 18 anos de sua vida…	**VIOLÊNCIA NA COMUNIDADE** As próximas perguntas são sobre a frequência com que **VOCÊ** viu ou ouviu certas coisas na sua **VIZINHANÇA OU COMUNIDADE** (não na sua casa ou na TV, rádio ou em filmes). Nos primeiros 18 anos da sua vida…
7.1 [V4]	*Did you see or hear someone being beaten up in real life?* *Many times* *A few times* *Once* *Never* *Refused*	Você viu ou ouviu alguém sendo espancado (na vida real)? [ ] Muitas vezes [ ] Poucas vezes [ ] Uma vez [ ] Nunca [ ] Não quis responder	Você viu ou ouviu alguém sendo espancado (não na sua casa ou na TV, rádio ou em filmes)? [ ] Muitas vezes [ ] Poucas vezes [ ] Uma vez [ ] Nunca [ ] Não quis responder/Não quero responder
7.2 [V5]	*Did you see or hear someone being stabbed or shot in real life?* *Many times* *A few times* *Once* *Never* *Refused*	Você viu ou ouviu alguém sendo esfaqueado ou levando um tiro (na vida real)? [ ] Muitas vezes [ ] Poucas vezes [ ] Uma vez [ ] Nunca [ ] Não quis responder	Você viu ou ouviu alguém sendo esfaqueado ou levando um tiro (não na sua casa ou na TV, rádio ou em filmes)? [ ] Muitas vezes [ ] Poucas vezes [ ] Uma vez [ ] Nunca [ ] Não quis responder/Não quero responder
7.3 [V6]	*Did you see or hear someone being threatened with a knife or gun in real life?* *Many times* *A few times* *Once* *Never* *Refused*	Você viu ou ouviu alguém ser ameaçado(a) com uma faca ou arma de fogo (na vida real)? [ ] Muitas vezes [ ] Poucas vezes [ ] Uma vez [ ] Nunca [ ] Não quis responder	Você viu ou ouviu alguém ser ameaçado(a) com uma faca ou arma de fogo (não na sua casa ou na TV, rádio ou em filmes)? [ ] Muitas vezes [ ] Poucas vezes [ ] Uma vez [ ] Nunca [ ] Não quis responder/Não quero responder
**8**	***EXPOSURE TO WAR/COLLECTIVE VIOLENCE***	**EXPOSIÇÃO A GUERRA/VIOLÊNCIA COLETIVA**	**VIOLÊNCIA COLETIVA**
	*These questions are about whether* ***YOU*** *did or did not experience any of the following events when you were a child. The events are all to do with collective violence, including wars, terrorism, political or ethnic conflicts, genocide, repression, disappearances, torture and organized violent crime such as banditry and gang warfare. When you were growing up, during the first 18 years of your life…*	As próximas perguntas são sobre se **VOCÊ** vivenciou ou não alguma dos seguintes acontecimentos quando você era criança. Todos eles estão relacionados com violência coletiva, incluindo guerras, terrorismo, conflitos políticos ou étnicos, genocídio, repressão, desaparecimentos, tortura e crime organizado violento, como bandidagem e guerra de gangues. Durante o período de crescimento, nos primeiros 18 anos de sua vida…	As próximas perguntas são sobre se **VOCÊ** vivenciou ou não algum dos seguintes acontecimentos quando você era criança. Todos eles estão relacionados com violência coletiva, incluindo guerras ou tiroteios, terrorismo, conflitos políticos ou étnicos, genocídio, repressão ou toque de recolher, desaparecimentos, tortura e crime organizado violento, como bandidagem, tráfico de drogas e guerra de gangues. Nos primeiros 18 anos da sua vida…
8.1 [V7]	*Were you forced to go and live in another place due to any of these events?* *Many times* *A few times* *Once* *Never* *Refused*	Você foi forçado a ir viver em outro lugar devido a algum desses acontecimentos? [ ] Muitas vezes [ ] Poucas vezes [ ] Uma vez [ ] Nunca [ ] Não quis responder	Você foi forçado a ir viver em outro lugar devido a algum desses acontecimentos? [ ] Muitas vezes [ ] Poucas vezes [ ] Uma vez [ ] Nunca [ ] Não quis responder/Não quero responder
8.2 [V8]	*Did you experience the deliberate destruction of your home due to any of these events?* *Many times* *A few times* *Once* *Never* *Refused*	Você vivenciou a destruição proposital da sua casa devido a algum desses eventos? [ ] Muitas vezes [ ] Poucas vezes [ ] Uma vez [ ] Nunca [ ] Não quis responder	Você vivenciou a destruição proposital da sua casa devido a algum desses eventos? [ ] Muitas vezes [ ] Poucas vezes [ ] Uma vez [ ] Nunca [ ] Não quis responder/Não quero responder
8.3 [V9]	*Were you beaten up by soldiers, police, militia, or gangs?* *Many times* *A few times* *Once* *Never* *Refused*	Você foi espancado por soldados, policiais, milicianos ou gangues? [ ] Muitas vezes [ ] Poucas vezes [ ] Uma vez [ ] Nunca [ ] Não quis responder	Você foi espancado por soldados, policiais, milicianos, gangues ou traficantes de drogas? [ ] Muitas vezes [ ] Poucas vezes [ ] Uma vez [ ] Nunca [ ] Não quis responder/Não quero responder
8.4 [V10]	*Was a family member or friend killed or beaten up by soldiers, police, militia, or gangs?* *Many times* *A few times* *Once* *Never* *Refused*	Algum familiar ou amigo(a) foi morto ou espancado por soldados, policiais, milicianos ou gangues? [ ] Muitas vezes [ ] Poucas vezes [ ] Uma vez [ ] Nunca [ ] Não quis responder	Algum familiar ou amigo(a) foi morto ou espancado por soldados, policiais, milicianos, gangues ou traficantes de drogas? [ ] Muitas vezes [ ] Poucas vezes [ ] Uma vez [ ] Nunca [ ] Não quis responder/Não quero responder
			Para terminar, gostaríamos de saber se até os seus 18 anos, você vivenciou algum outro evento traumático que não foi mencionado durante esta entrevista? [ ] Não [ ] Sim Qual(is)? __________________________________________ __________________________________________ __________________________________________

### Procedimentos Éticos

A autorização para conduzir o processo de adaptação transcultural do ACE-IQ foi requerida ao coordenador de prevenção de violência da OMS, e concedida via correio eletrônico.

O projeto de adaptação transcultural do ACE-IQ foi aprovado pelo Comitê de Ética e Pesquisa do Centro de Ciências da Saúde da UFES (Cepe/Ufes 2.141.917). Todos os participantes assinaram o termo de consentimento livre e esclarecido.

## RESULTADOS

A primeira e a última versão do questionário estão apresentadas no Quadro, juntamente com a versão original do instrumento, em inglês, disposta na primeira coluna. A versão final do EAI-QI está disponível no site da OMS: https://www.who.int/publications/m/item/adverse-childhood-experiences-international-questionnaire-(ace-iq)

Apenas três dos 43 itens de ambas as retraduções, quando comparadas ao instrumento original pelo comitê de especialistas, foram consideradas “muito alteradas” ou “completamente alteradas”. As discrepâncias encontradas ocorreram nas categorias de raça/cor autodeclarada (item C4), escolaridade (C5) e ocupação (C6), e foram resolvidas pelo R3, de acordo com os termos padronizados pelo Instituto Brasileiro de Geografia e Estatística (IBGE). Essas diferenças já haviam sido identificadas nas duas traduções iniciais. Ainda em relação às retraduções, os demais itens foram considerados pelos especialistas como “inalterados” ou “pouco alterados”, tendo sido considerada mais adequada a R2 em relação à R1, quanto à correspondência à versão original. Todos os itens foram revistos e, quando necessário, ajustados pelo R3.

Na avaliação global do questionário, o IVC foi correspondente a 1,0 em quase todos os quesitos de julgamento pelo comitê de especialistas, considerando a correspondência da S1 com a versão original em relação a pertinência/importância, clareza, vocabulário, objetividade, aplicabilidade ao contexto brasileiro e sequência instrucional dos itens. Dos 43 itens, apenas dois (C4 e A6) apresentaram o IVC < 0,7 (Tabela), sendo ajustados pelo R3, tanto na RS1 quanto na Q1. No item C4, os especialistas indicaram a inclusão da opção de resposta “outras”, considerando que algumas pessoas podem não se identificar com as alternativas apresentadas. No item A6, referente ao abuso sexual, foram apresentados pelos especialistas diversos formatos de reescrita do item, para frisar a ideia de que o respondente foi “forçado” a algo que não quisesse; no entanto, quando a questão original foi colocada em pré-teste, não foram observadas dificuldades de interpretação e, portanto, as sugestões de modificação feitas pelo comitê de especialistas não foram acolhidas. No geral, 73% dos quesitos avaliados receberam a pontuação máxima no IVC (1,0); em 21% o IVC foi 0,87; e em 4%, 0,75, sendo considerado adequado um valor de IVC > 0,8^33^. Apesar dessa pontuação satisfatória, todas as sugestões recomendadas pelos especialistas foram consideradas e, quando possíveis e pertinentes, incorporadas ao Q1.

**Tabela:** Itens do questionário que apresentaram quesitos com IVC inferior a 0,7 na avaliação do comitê de especialistas.

Quesitos avaliados	Item C4	Item A6	% IVC > 0,8^a^
	IVC	IVC	
Correspondência a versão original	0,62	0,62	95
Pertinência/importância	0,75	0,87	93
Clareza	0,37	0,62	93
Vocabulário	0,50	0,87	93
Objetividade	0,62	0,87	93
Aplicável ao modelo brasileiro	0,50	1	95
Sequência instrucional	0,75	1	98

IVC: índice de validade de conteúdo.

a Percentual de todos os cálculos de IVC nos 43 itens do questionário separado por quesito avaliado pelo comitê de especialista.

### Pré-teste 1

No primeiro pré-teste, conduzido com 11 participantes, os itens P3 e V2 apresentaram as menores médias de compreensão (2,72), avaliadas pela escala verbal de três pontos.

O item P3, que avalia a não provisão de alimentação adequada, foi interpretado erroneamente por alguns entrevistados como sendo devido à falta de condições financeiras dos pais ou responsáveis, porém a negligência física envolve o não provimento de condições básicas ao desenvolvimento infantil (alimentação, vestuário, assistência médica) na ausência de adversidade econômica^37^. Interpretação semelhante ocorreu no item P5 (média 2,82), no qual além da dificuldade financeira, foi aventada a impossibilidade de acesso e/ou a indisponibilidade local de escolas.

O item V2 foi resolvido com a inserção da instrução para que fosse escolhida apenas uma alternativa, referente à forma mais frequente de *bullying*, pois os participantes tendiam a marcar mais de uma resposta.

O item A3 foi discutido entre os pesquisadores após indicação dos participantes, no Pré-teste 1, de que a expressão *“*deu tapas*”* (dentre todas as outras formas de agressão) poderia não representar agressão física grave ou, ainda, que tais situações descritas no item pudessem ser entendidas como uma forma de correção disciplinar, e não como abuso físico. Esse resultado levou a ponderações e recomendações sugeridas pelos pesquisadores (ver “Limitações do instrumento e recomendações”).

Nos itens de A5 a A8, todos os entrevistados que sofreram abuso sexual manifestaram dúvidas em relação ao que estava sendo avaliado, isto é, se o abuso se restringia ao ambiente domiciliar e, consequentemente, foi cometido por membro(s) da família, ou se incluía qualquer abuso sexual sofrido. Isso se deu porque, nessa seção, há uma mudança abrupta e sem explicação prévia no modo de formulação das perguntas, em relação às seções anteriores. Estas eram precedidas da frase “Algum de seus pais ou responsáveis ou alguém que morava na sua casa…?”, e, na seção relativa a abuso sexual, a frase introdutória passou a ser “Alguém…”, sem que houvesse nenhuma especificação do agressor. Segundo as orientações para a utilização de ACE-IQ^34^, esses itens avaliam o abuso sexual infantil cometido por qualquer pessoa – familiares, conhecidos ou desconhecidos da criança. Para resolver essa dificuldade e esclarecer as possíveis dúvidas, optou-se por modificar a pergunta introdutória, de forma que as quatro questões dessa seção passaram a ser precedidas pela frase “Algum de seus pais ou responsáveis ou alguém que morava na sua casa ou algum conhecido ou desconhecido…”, sendo, ainda, incluído um subitem em cada questão, para distinguir se os abusadores eram ou não moradores da casa (ver “Limitações do instrumento e recomendações”).

Os participantes do Pré-teste 1 fizeram algumas sugestões: 1) trocar a ordem do item C5 de “Qual é o seu mais alto nível de escolaridade?” para “Qual o seu nível de escolaridade mais alto?”; 2) deixar apenas uma opção de escolha para a situação de trabalho nos últimos 12 meses (C6), apesar de terem demonstrado dificuldade em escolher apenas uma alternativa de resposta; 3) ajustar a instrução, antecedendo a seção de violência doméstica (F6-8), retirando a palavra “coisas” e a expressão “não necessariamente com você”; 4) trocar a palavra “atingido” por “agredido” em F8; e 5) retirar a expressão “exposição a guerra” e incluir os termos “tiroteios e tráfico de drogas” nas instruções que antecedem as questões sobre violência coletiva (V7-10).

Foi incluído, ainda, um último item no questionário – “Durante o período de crescimento, nos primeiros 18 anos de sua vida, você vivenciou algum outro evento traumático que não foi mencionado durante a entrevista?” – e, em caso afirmativo, um espaço aberto para resposta (ver “Limitações do instrumento e recomendações”).

### Pré-teste 2

Todos os itens adaptados e as modificações sugeridas a partir dos resultados do Pré-teste 1 objetivaram empregar termos coloquiais utilizados no contexto brasileiro, de mais fácil compreensão pela população geral, dando origem a uma versão modificada do questionário (Q2), que foi avaliada no Pré-teste 2. A partir da leitura de cada item pelos participantes e do relato de sua compreensão, sugestões de modificações do texto foram solicitadas quando o conteúdo era diferente do original ou ofertadas por elas, quando julgavam que a questão poderia ser melhor formulada.

Após o Pré-teste 2, decidiu-se modificar parte dos itens P3 (“…mesmo que pudessem facilmente oferecer um alimento para você?”) e P5 (“…mesmo que tivessem a obrigação de fazer isso?”) para melhor compreensão da questão (Quadro).

O item C6 foi testado de duas formas: 1) permitindo a escolha de todas as opções que correspondessem à situação de trabalho dos entrevistados nos últimos 12 meses; e 2) de apenas uma resposta, que representasse a principal atividade profissional/ocupacional. Embora a instrução do questionário original solicite apenas uma resposta, optou-se pelo uso de múltiplas alternativas considerando a dificuldade para escolher apenas uma resposta no Pré-teste 1, talvez refletindo a frequente multiplicidade de empregos no Brasil. Outras pequenas mudanças foram realizadas após o Pré-teste 2 e incorporadas à versão final do instrumento, apresentada no Quadro.

## DISCUSSÃO

A utilização de instrumentos padronizados de avaliação tornou-se uma prática necessária nas ciências da saúde, visando o aprimoramento do conhecimento científico. A sistematização da coleta de informações promove a redução de divergências, aumentando a qualidade da informação e, consequentemente, possibilitando a comparabilidade de resultados. Além disso, produz melhor embasamento para planejar políticas públicas e condutas clínicas^31^.

Os questionários estruturados são instrumentos amplamente utilizados em pesquisas epidemiológicas, em diversas áreas de interesse. Antes de se considerar o desenvolvimento de algum novo método de avaliação, faz-se necessária uma rigorosa revisão bibliográfica para a identificação dos instrumentos já existentes que permitam a avaliação desejada do objeto de estudo^33^. Em relação aos eventos traumáticos vivenciados na infância, é possível encontrar uma variedade de instrumentos disponíveis^24,36^. Ao identificar algum instrumento adequado aos objetivos do estudo que não esteja disponível no idioma da população a ser estudada, a melhor alternativa seria dar início à adaptação transcultural^38^.

A disponibilização do ACE-IQ para uso no Brasil, através da adaptação transcultural aqui descrita, possibilitará a utilização de um instrumento proposto pela OMS que permite a investigação de um número maior de EAI do que outros instrumentos em português, com o intuito de estabelecer avaliações estruturadas e produzir medidas comparáveis entre diversos países, com vistas à construção de perfis epidemiológicos da exposição a adversidades e eventos traumáticos na infância.

Neste artigo, apresenta-se a equivalência semântica do ACE-IQ da versão produzida em português vigente no Brasil. A adaptação transcultural é um processo minucioso e a equivalência semântica é considerada apenas uma de suas etapas^32^, embora não haja consenso sobre a forma mais adequada para a execução da adaptação transcultural e diversas abordagens metodológicas tenham sido propostas^30,31,32,39,40^.

A validade de conteúdo foi apresentada como parte da equivalência semântica, consistindo no julgamento sobre a representatividade e clareza de cada item do instrumento, segundo critérios pré-estabelecidos, avaliando-se, assim, a concordância entre os juízes sobre a adequação do conteúdo ao que o instrumento se propõe a medir^35,41^. Apesar de ser uma avaliação apontada como subjetiva por alguns autores^35,36^ é um processo frequentemente realizado nas ciências da saúde^42,43,44^, não dispensando, no entanto, a avaliação de outras medidas psicométricas^45^. Constatou-se boa equivalência semântica entre a versão em português e a original, através da validade de conteúdo, considerando-se a avaliação do comitê de especialistas e o cálculo do IVC e das médias de compreensibilidade durante os pré-testes.

Este estudo deve ser considerado à luz de algumas limitações. Os pré-testes foram realizados em amostras de voluntários não representativas da população geral adulta brasileira e, portanto, poderá haver variabilidade na compreensão dos itens ou expressões em amostras com distinta distribuição de idade, escolaridade ou classe socioeconômica, que poderá ser apreciada em estudos posteriores, quando o questionário produzido for aplicado em outras amostras. O instrumento original apresenta algumas expressões idiomáticas que não têm equivalência direta na língua portuguesa vigente no Brasil, tendo suscitado a exploração extensiva de alternativas aproximadas entre os respondentes e o comitê de especialistas. A avaliação da violência comunitária e coletiva envolve múltiplos aspectos, geralmente dependentes do contexto sociocultural da população investigada e socioeconômico/político da região/país, havendo itens que não são relevantes para o contexto da população brasileira, como a exposição a situações de guerra, terrorismo, genocídio, conflitos políticos e étnicos, entre outras. Por outro lado, a investigação de violência comunitária, extremamente relevante em estudos brasileiros, associada à presença de tráfico de drogas e de ações milicianas e/ou policiais, especialmente em regiões socialmente mais vulneráveis, com a exposição direta a brigas de gangues e balas perdidas, foi incluída no EAI-QI como violência coletiva, havendo, portanto, uma convergência desses contextos na avaliação de alguns estratos da população brasileira.

### Limitações do Instrumento e Recomendações

O instrumento adaptado neste estudo, assim como a maior parte dos instrumentos que avaliam EAI, foi projetado para a investigação da exposição a EAI em adultos (idade ≥ 18 anos), podendo ocorrer vieses de memória na coleta de informação. Além disso, indivíduos expostos a violências de maior gravidade têm risco aumentado de óbito e, portanto, o espectro mais grave da exposição não poderá ser capturado por essa forma de avaliação.

O ACE-IQ incorporou, junto à avaliação de EAI já estudada, a investigação de outras exposições, como *bullying* e violência coletiva ou na comunidade. Apesar dessa maior abrangência na identificação de EAI, de grande utilidade em pesquisas epidemiológicas, o instrumento apresenta limitações em sua forma de avaliar algumas das EAI. Assim, é pertinente apresentar algumas considerações e recomendações julgadas relevantes, tanto para o aprimoramento do instrumento, como para guiar a sua utilização, análise e interpretação de resultados em estudos que utilizam o EAI-QI. São elas:

1. Idade: todas as questões são precedidas por uma frase introdutória determinando que sejam consideradas apenas as experiências que tenham ocorrido “durante o período de crescimento, até os 18 anos de idade”. A exposição a EAI pode ter impacto deletério diferencial dependendo da idade de ocorrência. As crianças pequenas têm maior fragilidade física, são mais impotentes diante da violência, são mais vulneráveis ante a negligência e têm menor compreensão do que se passa à sua volta. Por outro lado, os adolescentes tendem a sofrer maus-tratos com maior intensidade e gravidade do que as crianças menores, mas, também, podem ser capazes de apresentar algum grau de enfrentamento e/ou de esquiva. Assim, sugere-se que a investigação da ocorrência de EAI seja relativa a, pelo menos, dois períodos distintos, infância (até os 10 anos) e adolescência (dos 11 aos 18 anos). Além disso, a duração da exposição a EAI também tem implicação direta nas consequências negativas, devendo ser incluída no questionário.
2. Gravidade do abuso físico: na questão A3, estão juntas expressões que envolvem variadas intensidades de agressão física (desde “deu tapas” a “chutou” e “socou, surrou”), gerando dúvidas na resposta, já que algumas formas de agressão física leve podem ser culturalmente justificadas como métodos disciplinares. Uma tentativa de resolver essa dificuldade foi retirar a expressão “deu tapas”. O abuso físico de uma criança é definido como “o uso intencional da força física que resulta, ou tem uma alta probabilidade de resultar, em danos à saúde, à sobrevivência, ao desenvolvimento ou à dignidade da criança”^3,46^. Assim, sugere-se acrescentar subitens para classificar a intensidade das agressões (leve, moderada, intensa ou muito intensa) e produzir estimativas mais robustas acerca do abuso físico infantil.
3. Perpetração de abuso sexual: nos itens A5-A8, há uma mudança abrupta no formato das questões que investigam a ocorrência de abuso sexual, uma vez que todos os itens anteriores eram estritamente relativos ao ambiente familiar. Sugere-se a inserção de uma frase introdutória para esse módulo, esclarecendo que o objetivo desse módulo *é* investigar o abuso sexual ocorrido antes dos 18 anos e perpetrado por qualquer pessoa, conhecido ou não da vítima. Sugere-se, ainda, que seja acrescentado um subitem a todas as questões, identificando se o agressor *é* ou não morador da casa, já que essa informação tem relevância na compreensão do impacto dos diversos tipos de abuso sexual perpetrados contra crianças e adolescentes. Usualmente, o abuso sexual que ocorre dentro do ambiente doméstico tem caráter repetitivo sem que a criança tenha, inicialmente, consciência e discernimento acerca do abuso. Assim, o perpetrador, que deveria cuidar e proteger, tende a se beneficiar da relação de confiança e poder, aproveitando-se da vulnerabilidade, imaturidade e insegurança, para impor à criança ou ao adolescente, sob ameaças e/ou força física, a agressão sexual e o silêncio^47^. Fora do ambiente doméstico, o abuso sexual tende a ocorrer de forma eventual, e a vítima é refém do agressor somente durante a agressão per se. De qualquer modo, o abuso sexual é um fenômeno traumático com graves consequências e a identificação dos agressores pode ser fundamental para a implementa*ção de* estratégias preventivas, além de ter relevância clínica no manejo terapêutico das vítimas de abuso sexual.
4. *Bullying*: somente a forma mais frequente de *bullying* é investigada, desconsiderando a multiplicidade e a gravidade das agressões. Além disso, não é investigada a possibilidade de que a vítima seja também agressora, tendo em vista que crianças e adolescentes vítimas de EAI podem se tornar agressores, perpetuando um ciclo de abusos e naturalizando a violência na resolução de problemas. Assim, sugere-se que múltiplas alternativas de resposta sejam permitidas e que seja incluída a investigação de perpetração de *bullying*.
5. Outros tipos de EAI: a compreensão acerca das EAI modificou-se ao logo da história e, com isso, novas categorias foram definidas e exploradas na construção de novos questionários. Apesar do ACE-IQ apresentar maior variedade de EAI do que os instrumentos anteriores, algumas adversidades descritas em outros questionários não foram incluídas, como, por exemplo, a exposição a adversidades econômicas e a ocorrência de doenças físicas e/ou acidentes graves. Sugere-se, assim, que seja incluído um item final para identificar a ocorrência de outros eventos traumáticos não avaliados.

## CONCLUSÃO

O instrumento adaptado neste estudo apresentou boa equivalência semântica, tornando-se uma ferramenta disponível para a pesquisa epidemiológica no Brasil, permitindo a comparabilidade entre estudos nacionais e internacionais. Além disso, pode contribuir para o conhecimento científico acerca do impacto deletério das EAI na vida adulta da população brasileira, com grande relevância para o campo da saúde coletiva. A investigação sistemática da EAI tem, também, importância clínica, ao prover informações do contexto histórico traumático do paciente, possibilitando uma avaliação mais abrangente, permitindo diagnósticos mais precisos e tratamentos mais adequados e reduzindo, assim, custos do sistema de saúde, perda de produtividade e sofrimento e incapacidade pessoal.

Por fim, pode prover informações relevantes para fundamentar o planejamento e a formulação de políticas públicas de prevenção e intervenção, visando minimizar a exposição a experiências adversas e traumáticas na infância e adolescência.
